# Targeting the Interplay of Independent Cellular Pathways and Immunity: A Challenge in Cancer Immunotherapy

**DOI:** 10.3390/cancers15113009

**Published:** 2023-05-31

**Authors:** Angela Lauriola, Pierpaola Davalli, Gaetano Marverti, Spartaco Santi, Andrea Caporali, Domenico D’Arca

**Affiliations:** 1 Department of Biotechnology, University of Verona, 37134 Verona, Italy; angela.lauriola@univr.it; 2 Department of Biomedical, Metabolic and Neural Sciences, Via G. Campi 287, University of Modena and Reggio Emilia, 41125 Modena, Italy; pierpaola.davalli@unimore.it (P.D.); gaetano.marverti@unimore.it (G.M.); 3 Consiglio Nazionale delle Ricerche (CNR) Institute of Molecular Genetics “Luigi Luca Cavalli-Sforza”, 40136 Bologna, Italy; spartaco.santi@cnr.it; 4 IRCCS, Istituto Ortopedico Rizzoli, 40136 Bologna, Italy; 5 BHF Centre for Cardiovascular Science, University of Edinburgh, Scotland EH4 2XU, UK; acaporal@exseed.ed.ac.uk

**Keywords:** immunotherapy, cancer, immunotherapy-challenges, immunotherapy-limitations

## Abstract

**Simple Summary:**

Although immunotherapy has improved the treatment and outcome of cancer patients, there are still limitations to face because most patients cannot receive lasting benefits. We believe it is urgent to discover new potential biomarkers and therapeutic targets for investigating personalized and less invasive anticancer treatments. In the present paper, we highlight: (i) the impact of ubiquitination and its reverse, de-ubiquitination, in orchestrating the immune response of the tumor microenvironment; (ii) selected clinical trials, which provide information on combination cancer immunotherapy and new immunomodulatory targets; (iii) current challenges in immunotherapy, including imaging technologies and ROS-based immunotherapies, as well as immunotherapy side effects. Finally, the major outstanding questions in cancer immunotherapy are also presented, and directions for future research are described.

**Abstract:**

Immunotherapy is a cancer treatment that exploits the capacity of the body’s immune system to prevent, control, and remove cancer. Immunotherapy has revolutionized cancer treatment and significantly improved patient outcomes for several tumor types. However, most patients have not benefited from such therapies yet. Within the field of cancer immunotherapy, an expansion of the combination strategy that targets independent cellular pathways that can work synergistically is predicted. Here, we review some consequences of tumor cell death and increased immune system engagement in the modulation of oxidative stress and ubiquitin ligase pathways. We also indicate combinations of cancer immunotherapies and immunomodulatory targets. Additionally, we discuss imaging techniques, which are crucial for monitoring tumor responses during treatment and the immunotherapy side effects. Finally, the major outstanding questions are also presented, and directions for future research are described.

## 1. Introduction

Cancer is considered one of the most alarming diseases for the human population in the world, although the mortality rate has continuously decreased since 1991 [1]. Cancer immunotherapy has emerged as a relevant therapeutic approach, allowing it to transform cancer treatment [2,3]. The percentages of patients responding to cancer immunotherapy are higher than those responding to genome-driven oncology treatments, but they are limited to only a patient’s subset [4]. Immune-based options or the progression of combined therapy based on cancer immunotherapies with other treatments face resistance to monotherapy with conventional immunotherapeutic modalities. Therefore, it is compelling to discover new potential biomarkers and therapeutic targets for investigating novel and feasible anticancer treatments [5].

The immune system plays complex and dynamic roles in cancer onset/progression by showing host-protecting and tumor-sculpting actions [6]. Although tumors continually elicit new questions, they generally evade immunotherapy through two main strategies: eluding immune recognition and generating an immunosuppressive tumor microenvironment (TME). TME is a complex network composed of several cell types and extracellular components, including extracellular matrix and secreted mediators (growth factors, cytokines, and chemokines). The interactions between tumor epithelium and the cells present and recruited to TME can positively and negatively regulate tumor genesis/progression/metastasis. TME is also involved in clinical intervention and treatment outcomes that are strictly related to therapeutic efficacy and long-term prognosis. The growing knowledge of TME’s role in regulating specific immune cells prompts the development of new therapeutic approaches, especially considering that TME cells are generally free from major genetic mutations compared to the tumor epithelium. Identifying TME immunosuppressive signals is a promising therapeutic strategy to reshape TME into an anticancer environment [7,8].

Recently, remarkable progress has been made at the molecular level in understanding the impact of ubiquitination, a type of post-translational modification, on tumor immune surveillance [9,10].

In the present paper, we highlight the impact of ubiquitination and its reverse, de-ubiquitination, in orchestrating the immune response of TME. Ubiquitination governs PD-1/PD-L1 expression in tumors, thus resulting in an associated clinical response to anti-PD-1/PD-L1 therapy in cancer patients. Targeting ubiquitination could be a potential strategy to potentiate immunotherapeutic effects in cancer patients [11,12].

Moreover, we emphasize various molecular imaging techniques and probes crucial for monitoring tumor responses to treatments. These techniques combine biomedical imaging and molecular biology to make visible and quantify the spatiotemporal distribution of immune-related tumor responses and progression for diagnostic and therapeutic applications [13,14]. We also indicate the most commonly used imaging techniques to morphologically characterize secreted exosomes upon isolation from CAR-T cells during anti-cancer immunotherapy [15,16].

Finally, we provided information on combined cancer immunotherapy in different tumor types and described their side effects. Clinical trials are reported based on the combination of ICI therapy with chemotherapy, PARP inhibitors, radionuclides, vaccines, novel hormone therapies, and CART-cell immunotherapy. Furthermore, owing to the intense development of ROS-based cancer immunotherapy, we underline ROS-modulating approaches in TME, which are proposed or applied in synergy with current immunotherapeutic procedures.

## 2. Oxidative Stress in Cancer Immunotherapy

Despite the great success of immunologic treatments in cancer therapy, too few patients with solid tumors show long-lasting beneficial effects, essentially caused by immunosuppressive mechanisms. Low response rates, primary or acquired resistance, and toxicity are the main challenges in obtaining satisfactory cancer immunotherapy. Due to patients’ resistance to monotherapy with conventional immunotherapeutic modalities, current studies have designed immune-based options or progressions of combination therapy based on cancer immunotherapies with other treatments. Several strategies combining immune checkpoint inhibitors (ICIs), tumor and/or immune-adjuvants, and/or cancer immunotherapy based on reactive oxygen species (ROS) have been demonstrated to inhibit primary and metastatic malignancies, as well as their relapse, and cause limited immune-related adverse events, even in the presence of tumor heterogeneity and multiple drug resistance [3,17].

### 2.1. Oxidative Stress in the Tumor Microenvironment

The immunosuppressive mechanisms in solid tumors are strictly related to the oxidative stress condition (OS) induced by oxygen-centered oxidants, namely ROS. It is well known that ROS are both agents and mediators of OS in multifaceted aspects of physiological and pathological processes, including innate and adaptive immunity. A high ROS steady-state is a unique feature of tumors, generally due to defects in oxidative metabolism and the accumulation of labile iron [18,19].

Although tumors can produce high levels of antioxidants, they must face the OS condition. Diverse tumor microenvironment (TME) cell types, including cancer-surrounding cells such as innate and adaptive immune cells, contribute to generating OS. OS is involved in immune regulation and response by directly acting on immune cells and their mutual interactions. These cells respond to ROS stimulation in a coordinated way [18,20]. Tumor-associated macrophages, myeloid-derived suppressor cells, and regulatory T cells (Tregs) are among the TME components that can produce excessive ROS and cause resistance to cancer immunotherapy through the apoptosis-related factor ligand pathway [19,21,22]. Overall, the complex interactions of cells in TME can determine the features and competence of immune TME against tumor cells. To overcome cancer disease, it is essential to target OS in all the cell types of TME [21,23].

### 2.2. Dynamic ROS Levels in Tumor Immune Microenvironment

Different ROS species and concentrations exert different roles and effects depending on the characteristics of the immune TME in which they are generated. Low to moderate ROS levels are required as signaling molecules that can arouse the immune system. Indeed, they stimulate T cell activation/differentiation and regulate tumor immune-response processes. Among other things, ROS stimulate the release and presentation of tumor-specific antigens (TSA) on the surface of cancer cells and their recognition by the immune system. ROS enhance the presentation of cancer cells’ major histocompatibility complex class antigen (MHC). MHC interacts with T cell receptors and triggers the immune response against cancer cells [19,24]. Sufficient immunogenicity of cancer cells in solid tumors, which is mainly due to TSA number, is an essential requirement for the immune system to eliminate cancer cells in the presence of cancer immunotherapy. Low expression of MHC makes cancer cells escape recognition from the immune system. Moderate ROS levels regulate the activation, proliferation, phenotypic differentiation, and survival of T cells, particularly cytotoxic leucocytes (TILs), which possess a specific cell-killing activity. Upon ROS stimulation, an increased number of immune cells such as dendritic cells (DCs) and TILs infiltrate TME. ROS triggers DC differentiation from monocyte precursors or hematopoietic cells. Immature DCs have migratory ability, while mature DCs can arrest diverse antigens and present them to T cells to initiate the immune response against TSA [19,21]. The infiltration turns the tumor from a low immunogenic state (“immunologically cold tumor”) to a highly immunogenic state (“immunologically hot tumor”) [25]. Moreover, moderate ROS levels in TME inhibit immune regulatory cells, such as Tregs; thereby, ROS prevents immune escape and diminishes immune suppression.

High ROS levels in TME are cytotoxic, can provoke stress responses, and trigger anti-tumorigenic signaling by initiating OS-induced cancer cell death. ROS promote T cell apoptosis by upregulating Fas and downregulating anti-apoptotic Bcl2 expression [26]. Some immune cells adapt to prolonged OS and undergo dysfunction to generate immunosuppressive signals in TME. This condition can suppress the immune response against cancer cells. The DC’s ability to present TSA to T cells is prevented, and T cell attack and interaction of T cell receptors with MHC are inhibited. Immunosuppressive TME can lead to immune evasion of cancer cells and promote cancer progression and metastasis [19,27].

### 2.3. ROS in Cancer Immunotherapy

The ROS modalities of action in signaling events and the immunological response of TME have suggested the possibility of ROS exploitation in immunotherapeutic treatments. Furthermore, an increasing number of preclinical and clinical studies have revealed that the modulation of ROS levels in TME can both control tumor progression and reverse immunotherapy resistance, thus exerting anti-tumor effects [21,22,27,28].

A great number of therapeutic strategies in tumor immunotherapy harness the dynamic ROS balance in TME because solid tumors frequently lack T cell infiltration and, in addition, ROS levels in TME are frequently heterogeneous. Multiple mechanisms, such as deficiency in antigen presentation and highly immunosuppressive TME, can hamper the induction of anticancer immunity obtained by ROS-generating therapies alone [17,19,29]. On the other hand, the unique administration of ICIs by anti-PD-1 is highly dependent on T cell activation in TME. However, it generates only 10–30% response rates when solid tumors exhibit immunosuppressive TME [17,21].

ROS-generating therapy can promote the conversion of immunosuppressive “cold” tumors to “hot” tumors and increase the sensitivity of TME to immunotherapeutic treatments. Although there is no clear conclusion about how to efficiently enhance the therapeutic outcomes of ICIs, reversing immunosuppression and increasing tumor-infiltrated T cell numbers are two important points that have been commonly accepted [20,22,30]. Challenging aspects need to be considered for an efficacious combination of immunotherapeutic treatments and ROS-generating therapies because ROS concentration in TME is critical. Whether ROS augment tumorigenesis or lead to apoptosis depends on intracellular ROS levels. Enhanced ROS levels can be a double-edged sword in the immunomodulation process.

The immunostimulatory and immunosuppressive ROS effects must be considered in evaluating the anticancer modalities. It is important to evaluate the impact of molecules and drugs used for OS control on PD-L1 expression and function [31]. ROS detrimental effects on anti-cancer immunity have been reported since ROS can drive macrophage polarization to immunosuppressive types, promote PD-L1 expression, and attenuate the efficacy of ICIs therapy. Additionally, ROS can deactivate T cells and inhibit the occurrence of immunogenic cell death (ICD) [24,32]. It has been suggested that patients who do not respond to immunotherapeutic treatments with PD-1 antibodies may have high ROS and hypoxia levels in TME, which result in compromised T cell responses. Furthermore, it has been demonstrated that adoptive immunotherapy with CAR T cells (CAR-T therapy) can alter tumor metabolism, leading to glutathione depletion and ROS accumulation in tumor cells [23].

Furthermore, even the best results of the most efficient combined therapies cannot be generalized to different treatment modalities. Inhibition of the cellular antioxidant system interferes with ROS metabolism in cancer cells; however, given the multiple ROS roles and effects in TME, ROS scavenging interventions in combined immunotherapies have obtained controversial results. A fully competent immune system is required to maximize the anti-proliferative effect of vitamin C (Vit C) in diverse types of murine tumors. Vit C enhances the cytotoxic activity of Tils in CAR-T therapy and cooperates with ICIs therapy. This has provided a rationale for clinical trials combining ICIs with high doses of Vit C, as this antioxidant protection treatment can convert ROS into less reactive species [33,34,35].

However, multiple clinical trials with Vit C have yielded unsatisfactory results, suggesting that factors other than tumor stage are important in determining the antioxidant effects of antioxidants. ROS can increase PD-L1 expression, but ROS elimination does not allow the elimination of PD-L1 molecules. Furthermore, ROS scavenging is hazardous as it attenuates ROS-stimulatory effects on immune cells and causes adverse effects due to the redox imbalance created by antioxidant therapy [22]. It is more advisable to exploit the ROS feature of acting through tunable roles, which can be adjusted or adapted to the different functional needs of TME cells. The dynamic equilibrium of ROS levels could be shifted from detrimental to beneficial by modulating ROS species and their concentration in specific locations [26]. It is suggested to investigate temporospatial windows in which ROS modulation in the tumor tissues of the patients may sensitize and/or synergize with anti-cancer immunotherapy to inhibit tumor growth and metastasis [22,31].

Translational significance and clinical progress of therapeutic OS modulating strategies in combination with chemo/radio/immunotherapies have been outlined for different cancer types. Clinical trials show that the combination of ROS-producing therapies with immunotherapies allows the achieving of substantial and synergistic efficacy, particularly in the ongoing pre-clinical settings, to eradicate primary tumors as well as metastasis and could trigger immunological memory to prevent tumor recurrence [17,21,36].

### 2.4. ROS-Based Cancer Immunotherapy

**Figure 1:**
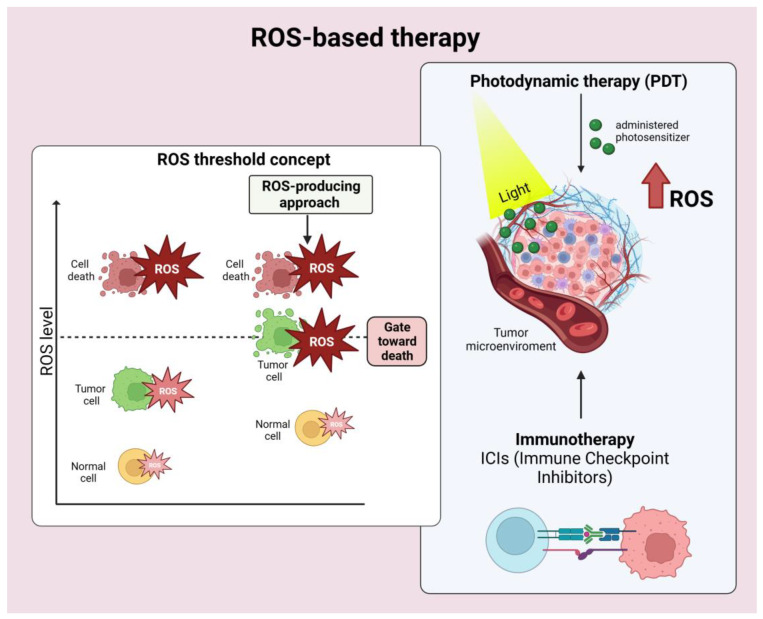
Different effects of photodynamic therapy (PDT) on ROS levels in normal and tumor cells. Normal cells have a basal level of ROS (radical oxygen species) required for cell survival and redox signaling. The production of ROS is elevated in tumor cells. When ROS reaches threshold levels, it is able to trigger cell death (the gate toward death). PDT is ROS-based therapy to increase ROS levels in tumor cells so they can reach the death threshold earlier. This therapy is enhanced in combination with immunotherapy.

The past few years have seen intense development of various strategies to power ROS-based cancer immunotherapy [17]. Here, we briefly underline examples of technical approaches to modulate ROS levels in TME that are proposed or applied in synergy with current immunotherapy procedures. Diverse exogenous modalities allow ROS delivery and modulation in cancer tissues without causing invasiveness to patients [17,31]. Tumor cells generally have higher ROS levels than normal cells. When ROS are increased through therapeutic approaches, ROS in tumor cells reach the death threshold earlier than in normal cells, resulting in a higher efficacy of anti-tumor therapeutics. These differences suggest the possibility of a ROS-generating therapy (Figure 1).

The ROS produced via ROS-generating therapies can directly and specifically damage cancer cells and/or induce inflammatory responses and ICD. ICD promotes DC maturation, which is essential in initiating adaptive tumor immune responses. Among the ROS-based therapies, radiotherapy (RT), photodynamic therapy (PDT), and sonodynamic treatment (SDT) are primarily utilized to increase ROS and ICD in combination with tumor immunotherapy, particularly with ICIs treatments. RT [37,38,39], PDT [40,41,42], and SDT [43] generate ROS through different physical modalities. These therapies can be dually applied to overcome limitations of efficacy, such as poor ROS generation and decreased oxygen levels in TME, which eventually occur with single ROS-based therapies. The dual therapy application produces higher OS and ICD, enhanced tumor immunotherapy, and can be further potentiated by the current ICIs treatments [17].

A different approach to modulating ROS levels in TME and to empowering immunotherapy utilizes various chemotherapeutics, including anthracyclines, platinum coordination complexes, alkylating agents, camptothecins, arsenic agents, and topoisomerase inhibitors, to increase ROS through mitochondrial ROS generation. However, although chemotherapies are currently combined with different immunotherapeutic modalities, they are often limited by insufficient ICD induction and cause adverse clinical effects [19,32].

Various herbal agents are being investigated to induce ROS-mediated ICD to realize advanced cancer therapy in combination with immunotherapic procedures [44,45,46]. ROS production obtained by natural products such as Naringenin, which is one of the most abundant flavonoids in diets, enhances MHC-I antigen cross-presentation in the anticancer immune response [26,36]. The flavonoid puerarin sensitizes anti-PD-L1 therapy by regulating the ROS level, which allows the reversal of immunosuppression in tumor models [47]. Differently, compounds such as the ROS generator napabucasin are well tolerated in combination with ICIs upon administration to patients with advanced cancers. Several patients have tolerated the treatment, showing prolonged disease stabilization and overall survival. The compound is activated by the intracellular antioxidant nicotinamide adenine dinucleotide phosphate (NAD[P]H): quinone oxidoreductase and generates sufficient ROS to cause cell damage and ICD. The drug overwhelms the anti-oxidant defenses of TME, also in tumors resistant to ICI treatment [48,49].

A further mechanism to increase ROS in tumors is obtained by targeting their high antioxidant systems. Antioxidants can effectively induce cell death when combined with exogenous and endogenous ROS inducers but often show unpredictable interactions with chemo-drugs [32]. Interestingly, the potential pro-oxidant role of pharmacological doses of Vit C has been successfully harnessed as a radio-sensitizer and a chemo-sensitizer in preclinical studies and early-phase clinical trials. This has suggested that it may also increase both the efficacy and benefits of ICIs [33,34]. Furthermore, Vit C affects multiple immune cell functions and can help overcome resistance to ICIs due to cytotoxic T-lymphocyte antigen 4 (CLTA-4) and programmed cell death ligand 1 (PD-L1/PD-1) inhibitors. Several in vivo and in vitro studies have proven that high doses of Vit C enhance cancer immunotherapies, while only a few clinical studies have been reported [50].

Antioxidants remain a growing challenge for clinicians due to their instability, limited bioavailability, poor solubility, and low selectivity, which limit their therapeutic uses in cancer. In addition, contradictory epidemiological evidence shows that high doses of Vit C cause harmful effects over a long administration period and even increase the risk of cancer. Indeed, Vit C also regulates mechanisms through which cancer escapes T cells’ immune response and resists ICIs [34].

To overcome the pharmacokinetic limitations and side effects of ROS-generating treatments, nanotechnology allows the delivery and accumulation of targeted drugs into TME, thus providing their maximal therapeutic activities. Nano-formulations of prodrugs can enhance cancer immunotherapy by simultaneously inducing antitumor immune responses and reversing local immunosuppression [51,52]. In addition, nanoparticles can direct ROS-regulating compounds to modulate ROS generation and elimination [53,54]. Currently, TME-responsive nanomedicine offers promising strategies to reduce adverse effects and enable the co-delivery of multiple immune modulators to yield synergistic cancer immunotherapy.

## 3. Ubiquitin Ligases in Cancer Immunotherapy

In recent years, remarkable progress has been made in understanding the impact of ubiquitination on tumor immune surveillance. Ubiquitination is a type of post-translational modification [9] in which a ubiquitin protein is bound to a substrate. Ubiquitin is covalently bound to target proteins of many soluble cytosolic and nuclear proteins. However, a subset of Cullin-RING ligase (CRL) complexes are recruited and activated locally at the cellular membrane [55]. The ubiquitin is covalently bound by the sequential actions of ubiquitin-activating (E1), ubiquitin-conjugating (E2), and ubiquitin-ligating (E3) enzymes. E3 ubiquitin ligases (E3), a family of about 700 enzymes, recruit the substrate and determine the specificity of ubiquitylation and subsequent proteasomal degradation or non-degradative process. A ubiquitin ligase can act as either a tumor promoter or a suppressor, depending on substrate specificity and biological function.

Increasing numbers of E3 ligases have been identified as regulators of tumor immune responses by enhancing anti-tumor immunity in cancer immunotherapy [10]. Many E3 ligases are expressed in different cell types of the immune system, where ubiquitination is involved in regulating innate and adaptive immune responses [56]. Targeting E3 ligases offers the possibility of improving anti-tumor immunity or developing new drugs to treat tumors in the future. Emerging evidence suggests that E3 ligases regulate tumor immune surveillance and can be used to boost anti-tumor immunity [57].

Immunotherapies consist of antibodies targeting immune checkpoint proteins (ICB), such as PD-1 (programmed cell death protein 1) on T cells and its ligand PD-L1 (programmed cell death 1 ligand 1) on antigen-presenting cells and tumor cells, that help tumor cells evade immune surveillance [58]. Recently, it has been demonstrated that PD-L1 is protected from ubiquitination-mediated degradation by binding with SGLT2 (sodium-glucose cotransporter-2). Indeed, the destruction of the interaction between SGLT2 and PD-L1 by the SGLT2 inhibitor canagliflozin allows the recognition of PD-L1 by Cullin3/SPOP E3 ligase for its proteasomal degradation [59]. Furthermore, PD-L1 was found to interact with the RNF125 E3 ligase, whose overexpression decreases the PD-L1 protein level by increasing its ubiquitylation. RNF125 E3 ligase suppresses tumorigenesis and growth in vivo and inhibits immune escape in head and neck squamous cell carcinoma (HNSCC) [60].

Since many studies have reported that tumor cells often become resistant to anti-PD-L1 therapy via immune evasion, the most crucial issue is to discover new therapeutic targets and potential biomarkers for cancer. In fact, current data suggest that not only ubiquitination but also its reverse, de-ubiquitination, plays a crucial role in orchestrating an immune response. In a recent study, the ubiquitin-specific protease 8 (USP8) has been shown to de-ubiquitinate PD-L1 in pancreatic cancer, demonstrating that USP8 intervention might increase PD-L1 blockade. Furthermore, it has been demonstrated that USP8 deficiency induced a time- and dose-dependent decrease in the PD-L1 protein level and increased the amount and function of tumor-infiltrated activated T cells [61]. Moreover, in colorectal cancer (CRC), a proteasome-associated deubiquitinating enzyme, USP14, was discovered to be an attractive target for CRC immunotherapy. Specifically, the knockdown of USP14 decreases indoleamine 2,3 dioxygenase 1 (IDO1) expression, abolishes suppression of cytotoxic T cells, and increases responsiveness to anti-PD-1 in a syngeneic MC38 mouse model [62].

The available ICB antibodies are proving ineffective in tumors with an immunosuppressive tumor microenvironment (TME), and there is an urgent need to find new targets to reprogram the suppressive TME. Recently, a large-scale in vivo CRISPR screen identified COP1, an E3 ubiquitin ligase, as a novel immune target regulating the TME of triple-negative breast cancer (TNBC). Silencing COP1 in cancer cells suppresses macrophage infiltration and enhances anti-tumor immunity [63]. Another study demonstrated the relationship between 13 E3s and immunological features in the TME of patients with lung adenocarcinoma (LUAD), showing a negative or positive correlation with most immune modulators and tumor-infiltrating immune cells (TIICs). Furthermore, they selected membrane-associated RING-CH-1 (MARCH1) as the inflamed TME in LUAD, suggesting that this E3 ligase is a novel and promising biomarker of immune status and immunotherapy efficacy in this type of tumor [64].

Additionally, it is worth noting that TME plays an essential role in the extracellular matrix (EMC), which is dynamically remodeled and regulated by various factors. In gastric cancer, it has been shown that TRIM44, an E3 ubiquitin ligase, regulates the stability of the LOXL2 protein, a key factor for crosslinking collagen and important for remodeling the ECM, thus regulating tumor immunity [65]. They suggest the TRIM44/LOXL2 complex as a potential biomarker for gastric cancer prognosis and as a novel immunotherapy target.

Recently, a new type of small molecule, named proteolysis targeting chimeras (PROTACs), has emerged for inducing ubiquitylation of the target protein. PROTAC are potent tools for the degradation of selective proteins in cancer, and they are a valuable approach for improving anti-tumor immunotherapy. PROTACs consist of two ligands connected by a linker. One ligand recruits and binds a substrate, while the other recruits an E3 ubiquitin ligase. Although only a few E3 ligases have been used in PROTACs technology, they have several advantages, including solubility and cell permeability. They are therefore promising against targets that are impossible or difficult to target. PROTACs have been described to enhance anticancer immunotherapy by regulating specific proteins, including IDO1, PD-L1, SHP2, HDAC6, HPK1, BCL-xL, BET, NAMPT, and COX-1/2. While the specific role of ubiquitination signaling in the immune defense is still unclear and the immunotherapies targeting ubiquitination are not fully understood, the interplay of the ubiquitin system and TME deserves more investigations in the future [12].

## 4. Imaging in Cancer Immunotherapy

Monoclonal antibody-based therapies targeting immune checkpoint inhibitors (ICIs) have shown promising results in the past decade. These therapies allow for the recognition of tumor antigens by the host’s T cells, generating an antitumor immune response that bypasses the host’s innate immunity [66,67]. Examples of well-known immuno-mechanisms include the CTLA-4/CD152 pathway in melanoma [66], the programmed cell death protein 1 (PD-1/CD279) pathway in various types of cancer such as melanoma, RCC, HNSCC, NSCLC, ovarian cancer, Merkel cell carcinoma, B-cell lymphoma, follicular lymphoma, and urothelial carcinoma [68,69], and the human epidermal growth factor receptor (EGFR) 2-directed monoclonal antibody in HNSCC and CRC [70].

Imaging techniques are crucial for monitoring tumor responses during treatment. These techniques include computed tomography (CT), positron emission tomography (PET), magnetic resonance imaging (MRI), and single photon emission tomography (SPECT). They use imaging biomarkers specific to molecular targets that can characterize immune-related tumor responses and progression.

The imaging tracers commonly used in immune oncology are labeled with different radiolabels, such as 18F for detecting abnormal glucose metabolism and inflammation sites, 15O for measuring blood flow, 11C for neuronal disorders, prostate cancer, and lung carcinomas, and 123I, 99mTc, 133Xe, 201Tl, and 111In for SPECT imaging. Specific radiolabeled tracers include 89Zr-atezolizumab for PD-1 and EGFR targets and 64Cu-DOTA for CTLA-4 antigen [13,14,71].

Despite the explosion of immune-oncological drugs in the past decade, this strategy has several limitations [72]. Firstly, the development of clinical imaging biomarkers for anti-cancer immunotherapy does not keep up with the speed of immunotherapy development. Secondly, the spatial resolution of clinical cameras/scanners depends on the radioisotopes’ positron range, which can vary from 1 to 4 mm Full Width Half Maximum (FWHM). FWHM is a commonly used parameter to describe the quality of an image; a lower FWHM value indicates a sharper image and, therefore, better spatial resolution [73]. Finally, the need to test new markers on animal models with different genetic makeup and immune systems than humans prevents a clear understanding of the tumor microenvironment at the cellular and subcellular levels [74].

CAR-T cell therapy is an emerging cell-based approach to immunotherapy that involves modifying T cells to express a chimeric antigen receptor (CAR) that can recognize specific antigens in cancer cells. These therapeutic cells can recognize CD19 on the tumor cell surface and release cytokines to kill the cancer cell in leukemia [75]. However, there are still many questions that need to be addressed regarding the efficacy and safety of CAR-T cell therapy, including low efficacy against solid tumors, CAR-T cell dysfunction or exhaustion, neurotoxicity, supra-physiologic cytokine production, and massive in vivo T cell expansion [76]. Recently, a correlation between an increase in the secretion of proinflammatory cytokines and a decrease in tumor cell growth was demonstrated using 2nd generation CAR-T cells against modified solid tumor cell lines from different tumors [77]. To better understand the molecular mechanisms underlying cytokine release in CAR-T cell therapy, exosomes induced via T cell receptor (TCR) stimulation have been found to play a pivotal role [15]. Exosomes are nano-sized extracellular vesicles secreted by most body cells and carry a range of functional molecules, including proteins, DNA, mRNAs, microRNAs, DNA–RNA hybrids, and lipids. Exosomes isolated from targeted CAR-T cells maintain most characteristics of the parental T cells, including surface expression of the CARs, CD63, and CD3. In addition, they can carry perforin, granzymes, and other effector molecules to directly kill cancer cells [78].

The most commonly used imaging techniques to morphologically characterize secreted exosomes include: Transmission Electron Microscopy (TEM) and Cryo-Transmission Electron Microscopy (Cryo-TEM), which provide high-resolution images of exosomes at the nanoscale level; Scanning Transmission Electron Microscopy (STEM) can provide elemental analysis of exosomes; Atomic Force Microscopy (AFM) can be used to analyze exosome surface topology and mechanical properties. Single-Molecule Localization Microscopy (SMLM) is a super-resolution microscopy technique that can provide high-resolution images of exosomes with a resolution of a few nanometers [79].

### 4.1. Electron Microscopy

**Figure 2:**
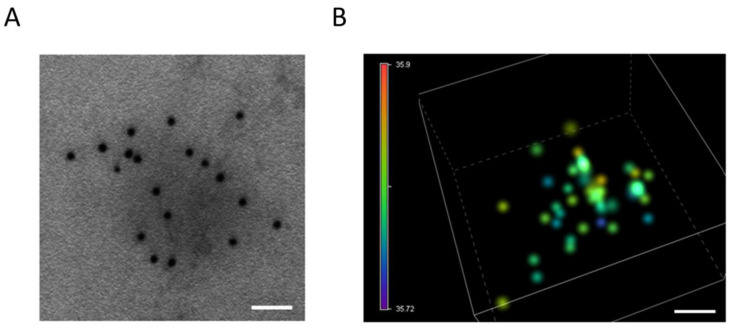
(**A**) Immuno-gold transmission electron microscopy analysis of extracellular vesicles isolated from plasma, immune-labeled with a 5 nm gold antibody. Scale bar, 20 nm. (**B**) 3D-STORM reconstruction with x, y, z coordinates of single-molecule localization within the TIRF plan for a 3D view of a plasma-recovered exosome immune-labeled with Alexa fluor 647. Single molecules are shown on a pseudo-color scale. Scale bar, 20 nm.

Electron microscopy is the gold standard technique for exosome characterization as it provides high-resolution imaging and morphology information (Figure 2A). Cryo-TEM is an extension of TEM that allows for the visualization of fresh exosomes while preserving their structure by avoiding fixation and dehydration. TEM is commonly used for exosome visualization and determining the diameter of the studied vesicles. The immuno-gold labeling of exosome samples in TEM and STEM is a method used to detect specific molecules present on the exosome surface and inside the cargo. However, this technique may reduce the ultrastructural preservation of the sample. In addition, the classic electron microscopy fixative, glutaraldehyde, is not compatible with immune labeling for most antibodies, and weaker fixation agents such as paraformaldehyde, which can maintain the binding of antibodies to the epitopes of the antigen, may not optimally preserve membrane structures. The main limitation of using electron microscopy for exosome characterization is the uneven and inconsistent distribution of exosomes onto grids, which makes it challenging to measure concentrations accurately [80].

### 4.2. Atomic Force Microscopy (AFM)

AFM can be used to study exosomes topographical morphology in their native conditions without extensive sample preparation and labeling [16]. It provides high-resolution 3D images of the exosome surface with very high resolution (<nanometer). However, it should be noted that exosomes can change their shape depending on their size and internal contents. AFM can also quantitatively measure the mechanical stiffness of exosomes using AFM-based force spectroscopy or the PeakForce tapping mode, which extracts a force-distance curve from a position on the exosome surface [81]. While this technique is labor-intensive and time-consuming, it can be used to distinguish between different subpopulations of vesicles based on their mechanical properties.

### 4.3. Single-Molecule Localization Microscopy (SMLM)

SMLM is an optical super-resolution microscopy technique that can visualize single molecules below the diffraction limit of light (Figure 2B). It uses the random activation of fluorescent probes with photoactivatable properties to localize events with high precision and reconstruct the acquired image at high spatial resolution. This phenomenon, often referred to as “blinking”, is sequentially repeated many times (from 2000 to 10,000 acquisition frames) until most molecules have been localized with a resolution of about 20 nm in xy and 50 nm in z [82].

At present, there are three primary methods to accomplish this: STORM (Stochastic Optical Reconstruction Microscopy), PALM (Photo-Activated Localization Microscopy), and PAINT (Point Accumulation for Imaging in Nanoscale Topography) are all Super-Resolution Microscopy (SMLM) techniques that use photoactivatable or photo-switchable fluorescent probes to achieve high resolution imaging. These techniques differ in how they achieve the activation and localization of a subset of molecules during the acquisition process. STORM and PALM use photoactivatable fluorescent probes that can be activated by light. In contrast, PAINT uses a fluorescent dye that transiently binds to the molecule of interest, resulting in a blinking signal that can be localized. Using an astigmatic lens and performing a mapping calibration with known-size fluorescent beads, it is possible to obtain 3D information from the molecule localization. Various blinking fluorophores are available, and multichannel setups are used for multi-parameter analysis and concentration measures.

However, this imaging technique has some limitations, including the absence of morphological information from the sample and the need for a specific buffer containing a reducing agent to switch the fluorophores to the dark state and an enzymatic oxygen scavenging system to eliminate oxygen from the environment. This is carried out to prevent permanent photo-bleaching, which is facilitated by oxygen and could compromise the quality and resolution of the final image. The use of inadequate imaging buffers can weaken the experiment’s final outcome, compromising the image’s quality and resolution through the suboptimal blinking behavior of the fluorophores.

## 5. Combination Cancer Immunotherapy and New Immunomodulatory Targets

### 5.1. Selected Clinical Trials Providing Information on ICI Combination with Chemotherapy in Different Tumor Types (Table 1)

The recent introduction of immune checkpoint inhibitors (ICIs) has prolonged the survival results for patients with different tumors, including those with advanced non-small cell lung cancer (NSCLC). The programmed cell death1/anti-programmed death ligand 1 (PD-1/L1) pathway, previously detected in a variety of malignant tumors, plays an important role in fighting tumors by regulating the function of autoimmunity.

However, when combined with chemotherapy, which can stimulate anticancer immune responses, pembrolizumab demonstrated response and survival improvements compared to chemotherapy alone, regardless of tumor PD-L1 production. In this regard, the first phase III RCT that evaluated an anti-PD1/PD-L1 treatment in combination with chemotherapy was the KEYNOTE 189 trial, which enrolled 616 patients with metastatic untreated non-squamous NSCLC randomized to receive carboplatin/cisplatin with pemetrexed plus either pembrolizumab or placebo every three weeks for four cycles [83].

Recently, pembrolizumab was also used in the Phase III KEYNOTE 407 trial that randomized 559 patients with untreated metastatic squamous NSCLC in a 1:1 ratio to receive either pembrolizumab or saline placebo, plus carboplatin and either paclitaxel or nab-paclitaxel [84]. Atezolizumab was also compared in association with platinum-pemetrexed versus chemotherapy alone in the first-line setting of 578 patients with stage IV nonsquamous NSCLC in the IMpower132 phase III trial. Of note, adding atezolizumab to chemotherapy led to a better ORR (47% vs. 32%) and a longer median duration of response (10.5 m vs. 7.2 m) compared with chemotherapy alone [85].

For first-line treatment of metastatic squamous NSCLC, atezolizumab was also investigated in the IMpower 131 phase III RCT in association with carboplatin plus paclitaxel/nab-paclitaxel, compared with carboplatin plus nab-paclitaxel alone [86]. Although breast cancer has historically been considered a non-immunogenic tumor, a small subset of breast cancers is immune-activated, with PD-L1 expression and/or TILs in the tumor microenvironment. Pembrolizumab or atezolizumab combined with chemotherapy increased median progression-free survival with the addition of immunotherapy, but the clinical benefit was modest. Only about 40% of triple-negative breast cancers are PD-L1+; not all PD-L1+ patients with advanced triple-negative breast cancer respond, and immunotherapy is not yet approved for advanced PD-L1-negative triple-negative breast cancer, HER2 + breast cancer, or ER + breast cancer.

Recent advances in the immunologic portrait of TNBC show the tumor is characterized by a unique microenvironment with higher levels of lymphocyte infiltration and PD-L1 expression than other BC subtypes and also has a more significant number of somatic mutations due to genomic instability, leading to the frequent presence of neoantigens [87]. Thus, the IMpassion130 trial provided strong confirmation that atezolizumab plus nab-paclitaxel (nab-PTX) improved both PFS (7.5 vs. 5.0 months) and 3-year OS (36.0 vs. 22.0 months) in the PD-L1 + TNBC than nab-PTX monotherapy [88]. In the IMpassion031 phase III study of atezolizumab in combination with neoadjuvant nab-PTX- anthracycline for early TNBC, the proportion of patients with pCR increased from 41% to 58% compared with the placebo group [89]. The combination of atezolizumab (A) + cobimetinib (C) + paclitaxel (P)/nab-paclitaxel (nP) also demonstrated favorable safety profiles in the presence or absence of chemotherapy [90].

The randomized phase II study by Loibl et al., 2019 [91], investigated durvalumab in addition to an anthracycline-taxane-based neoadjuvant therapy in early triple-negative breast cancer. A phase 1b–2 trial tested the combination of pembrolizumab plus trastuzumab in trastuzumab-resistant, advanced, HER2-positive breast cancer [92]. This combination gave an OR (15%, PD-1+; 0%, PD-1−). fatigue 21%. The treatment was safe, with activity and durable clinical benefit in PD-L1+/HER2+, trastuzumab-resistant, advanced BC patients.

Pancreatic cancer, which most commonly occurs as pancreatic ductal adenocarcinoma (PDAC), is a highly lethal cancer with the lowest reported 5-year survival rate (9% for all stages of the disease) among various cancers. Pancreatic cancer can suppress the host immune response directly or via immune cells in the tumor microenvironment. Resistance of PDAC to immunotherapy has been attributed to the poor intrinsic antigenicity of the tumor cells and defective antigen presentation, as well as a strongly immunosuppressive microenvironment enriched in MDSC and Treg cells [93]. The PD-1 immune checkpoint inhibitor pembrolizumab is recommended as second-line therapy for metastatic pancreatic cancer patients who have tested positive for mismatch repair deficiency, or MSI, and other agents are also undergoing preclinical/clinical trials as combination therapies. Weiss et al. [94] tested in metastatic PDAC gemcitabine + nab-paclitaxel + pembrolizumab in phase Ib/II, obtaining a 17 PFS: 9.1 months. OS: 15.0 months, 70.6% (12/17). Kamath et al. [95] reported a similar response rate for gemcitabine and ipilimumab in advanced pancreatic cancer compared with gemcitabine alone. Locally advanced or metastatic gemcitabine + ipilimumab Ib 21 ORR: 14% (3/21). PFS: 2.78 months. OS: 6.90 months, 76.2% (16/21; elevated ALT, diarrhea, mostly hematologic toxicity). Wainberg et al. [96] reported a similar response rate for gemcitabine, nab-paclitaxel, and nivolumab in advanced pancreatic cancer.

In gastric cancer, which is the fifth most common cancer and the third leading cause of cancer-related mortality, anti-programmed cell death protein 1 (PD-1) monoclonal antibodies have been approved in the chemorefractory setting for Asian patients and a subset of PD-L1-positive patients with advanced gastro-oesophageal adenocarcinoma.

Nivolumab (360 mg q3w) has shown efficacy and safety in a randomized phase II study in combination with S1(S)/capecitabine(Cape) plus oxaliplatin (OX) in patients with untreated, unresectable, advanced, or recurrent HER2-negative gastric/gastroesophageal junction adenocarcinoma, part 1 [97]. Nivolumab then blocks the protein, enabling the immune system to continue attacking the gastric, esophageal, or gastroesophageal junction cancer cells. This is particularly important since HER2-negative cancer cells can grow slower than HER2-rich cancer cells and are thus less likely to return or metastatize. It has been tested in CheckMate (649), a randomized, open-label, phase 3 trial in combination with chemotherapy [98]. By comparison, in KEYNOTE-059 Cohort 2, PD-L1-positive patients appeared to benefit more, with an ORR of 68.8% vs. 37.5%, by combining pembrolizumab alone or in combination with chemotherapy [99].

GBM is a locally immunosuppressive tumor, limiting the benefits that can be afforded by immunotherapy. GBM has a lower mutational burden than other primary tumors, such as metastatic melanoma and NSCLC, and is locally immunosuppressive. Thus, the efficacy of immunotherapy in GBM is limited by the tumor microenvironment and not suitable for immune therapies such as immune checkpoint inhibitors, which act in part through local T cell interactions and by the relative impermeability of the BBB; thus, some phase III trials failed to meet the primary end point of increased survival [100]. The combination of CT and immune checkpoint inhibitors is also being investigated for mCRPC since, by causing the release of antigens and stimulating the activity of cytotoxic T lymphocytes, CT shows an immunomodulatory effect on the tumor, regulating the composition and immunosuppressive pathways of the tumor microenvironment [101]. Cohort B of the phase II trial CheckMate 9KD evaluated the combination of docetaxel and nivolumab in 41 patients with mCRPC progression after second-generation hormonal therapy and CT-naïve. ORR was 36.8% in patients with measurable disease, PSA RR was 46.3%, and rPFS was 8.2 months [102]. Cohort B of the phase Ib/II KEYNOTE-365 trial analyzed the combination of docetaxel and pembrolizumab in 104 patients with the same characteristics. ORR was 18%, PSA RR was 28%, rPFS was 8.3 months, and OS was 20.4 months [103].

In a subsequent study, the efficacy and safety of the anti-PD-1 antibody pembrolizumab combined with the chemotherapy drug docetaxel and the steroid prednisone for patients with metastatic prostate cancer resistant to androgen deprivation therapy who had never received chemotherapy were also evaluated. The combination showed antitumor activity and manageable safety in this patient population [104]. The potential for synergistic activity through modulation of the microenvironment has been explored in a combination strategy of PD-1/PD-L1 inhibition with antiangiogenic therapy to overcome the modest activity of single-agent PD-1/PD-L1 immune checkpoint blockade in recurrent EOC [105].

### 5.2. Selected Clinical Trials Providing Information on ICI Combination with PARP Inhibitors in Different Tumor Types (Table 2)

HR and other DDR defects have recently been identified as potential predictive biomarkers of response to anti-PD1 therapy in various tumor types, including metastatic castration-resistant prostate cancer (mCRPC). Multiple independent early-phase clinical trials show that combining DDR-targeted therapies with ICI is safe and demonstrates encouraging antitumor efficacy. In cohort A of the KEYNOTE-365 study, 84 patients with mCRPC who progressed to docetaxel and second-generation hormonal therapies received treatment with olaparib plus pembrolizumab. Patients included had a PSA RR of 9%, an ORR of 8.3%, a rPFS of 4 months, and an OS of 14 months [106]. A phase II study has evaluated the combination of durvalumab and olaparib in 17 patients with mCRPC after progression to abiraterone and/or enzalutamide. rPFS was 16.1 months old, and 53% had a serological or radiographic response. Patients with alterations in DNA repair genes had a rPFS of 16.1 months and an ORR of 83% [107]. In a preliminary phase II clinical trial on metastatic castration-resistant prostate cancer with anti-CTLA-4 plus anti-PD-1, Sharma et al. report ORR of 25% and 10%, median rPFS of 5.5 and 3.8 months, and median OS of 19.0 and 15.2 months in pre- and post-chemotherapy patients [108]. A phase II study in breast cancer (BC) combined pembrolizumab with niraparib. It achieved an ORR of 47% in BRCA-mutated advanced or metastatic triple negative breast cancer (TNBC), compared with 21% in BRCA wild-type TNBC [109]. In a phase II trial, the addition of apatinib to camrelizumab documented a higher ORR (43.3%) in advanced TNBC than either apatinib or camrelizumab [110]. Given the immunological properties of PARPi, combining these drugs with ICIs may be an intriguing strategy to enhance the immune response to ovarian cancer (OC) cells.

Since approximately half of high-grade EOCs exhibit homologous recombination deficiency (HRD) in the DNA damage repair pathway, poly(ADP-ribose) polymerase (PARP) inhibitors have become a significant component of therapy for many EOC. HRD in EOC is also supported by somatic and germline alterations in BRCA1 or BRCA2 in up to 22% of high-grade serous carcinomas. The increased sensitivity to ICB in EOC tumors with HRD could depend on the fact that BRCA-mutant (BRCAm) tumors possess more mutations and CD8+ TILs than do BRCA wild-type (BRCAwt) tumors, which have a higher level of PD-L1 expression [111]. Combined PARP and ICI have yielded encouraging results in ovarian cancer, but predictive biomarkers are lacking.

In [112], the results of immunogenomic profiling and highly multiplexed single-cell imaging on tumor samples from patients enrolled in a Phase I/II trial of niraparib and pembrolizumab in ovarian cancer (NCT02657889) are reported. The TOPACIO/KEYNOTE-162 trial enrolled 62 patients in its phase I and II population with previously treated advanced or metastatic ovarian cancer considered platinum-sensitive to first-line therapy with subsequently acquired platinum resistance. Patients were treated with oral niraparib once daily for days 1 to 21 and intravenous pembrolizumab on day 1 of each 21-day cycle [113].

The open-label, investigator-initiated, phase 2 umbrella trial (NCT03699449) enrolled 70 patients with platinum-resistant ovarian cancer to receive combination therapy of durvalumab and olaparib based on homologous recombination deficiency (HRD) and programmed death ligand 1 (PD-L1) status determined by archival tumor sample assessment [114].

Many ICI plus single agent trials did not meet their co-primary endpoint of OS at the first interim analysis and/or did not demonstrate significant improvement in PFS or OS. Therefore, in recurrent EOC, the second-stage phase II study MEDIOLA trial was designed to test the triplet combination by adding bevacizumab 10 mg/kg intravenously every 2 weeks to olaparib and durvalumab in germline BRCAwt relapsed OC patients, which also includes a cohort of patients with non-germline BRCAm PSOC [115]. The phase 2 OPAL trial (NCT03574779) examined the triplet combination of dostarlimab, niraparib, and bevacizumab in patients with recurrent EOC. In the PROC cohort, which comprised mostly BRCAwt patients, the ORR was 17.9%, with 7 partial responses and 23 of 39 evaluable patients with stable disease [116], suggesting clinical activity in a population predicted to have poor responses to systemic therapies.

### 5.3. Selected Clinical Trials Providing Information on ICI Combination with Radionuclide in Different Tumor Types (Table 3)

Radioligand therapies represent another weapon available for possible combinatorial strategies against cancer, including mCRPC. In this regard, an escalating dosage of ipilimumab with or without radiotherapy was evaluated in patients with mCRPC [117].

A phase Ib trial has evaluated the combination of atezolizumab and Radium-223 in mCRPC. However, the clinical response was low, with an ORR of 6.8%, a PSA RR of 4.5%, and a rPFS of 3 months [118]. While RT-chemotherapy combination regimens are well established in oncology, this approach was largely unsuccessful in GBM until the introduction of temozolomide. The success of this combination has stimulated the search for other candidate drugs for concomitant use with RT in GBM, including ICI; however, many of these studies failed to meet their primary endpoint of increased survival. Nevertheless, a phase II trial is evaluating the safety and response to the treatment of durvalumab and/or bevacizumab in combination with radiotherapy in five cohorts of patients (*n* = 158) with newly diagnosed MGMT non-methylated and recurrent GBM (NCT02336165). Preliminary data from cohort A (*n* = 40, newly diagnosed GBM) revealed that durvalumab treatment with RT was well tolerated [119].

### 5.4. Selected Clinical Trials Providing Information on ICI Combination with Vaccines in Different Tumor Types (Table 4)

Most of the trials determining the role of vaccines in patients with advanced PDAC failed to show durable responses due to the tumor microenvironment, characterized by abundant mesenchymal origin fibroblasts, blood vessels, and tumor-infiltrating immune cells surrounded by extracellular matrix, thus facilitating cancer escape from immune surveillance. Nevertheless, several clinical trials have elucidated the clinical efficacy of vaccines in patients with severely progressed PDAC.

**Table 1:** Selected clinical trials providing information on ICI combination with chemotherapy in different tumor types.

Tumor Type	Combined Therapy	Anti-PD-1/PD-L1	Clinical Phase and Population	N of Patients	Result	Conclusion	NCT Number	Reference
LUNG non squamous	Pembrolizumab + platinum-pemetrexed	Pembrolizumab	III	616	Median OS: 22 m vs. 10.7 m (HR 0.56, CI95 [0.45 × 10^0.70^]) ORR: 47.6% vs. 18.9% Subsequnet ICI: 53.9%	Long-lasting benefit in first-line setting	KEYNOTE 189	[83]
LUNG advanced squamous NSCLC	Pembrolizumab/Carbo/Pacli or nab-Pac	Pembrolizumab	III; untreated metastatic	559	PFS (months) 6.4 HR (95%CI) 0.56 (0.45–0.70) OS (months) 15.9 HR (95%CI) 0.64 (0.49–0.85)	PFS and OS outcomes did not change based on taxane used	KEYNOTE 407	[84]
LUNG non squamous	Atezolizumab + platinum-pemetrexed	Atezolizumab	III	578	Median PFS:7.6 m vs. 5.2 m (HR 0.596, CI95 [0.494 × 10^0.719^], *p* < 0.0001) ORR: 46.9% vs. 32.2% Subsequnet ICI: 37.1%	Better ORR (47% vs. 32%) and longer median duration of response (10.5 m vs. 7.2 m) compared with chemotherapy alone. PFS benefit regardless of PD-L1 status	IMpower132	[85]
LUNG advanced squamous NSCLC	Atezolizumab + carboplatin-nab-paclitaxel	Atezolizumab	III	1021	Median OS: 14.2 m vs. 13.5 m, NS Subsequnet ICI: 43.2%	Prolonged median OS in the PD-L1 high subgroup (23.4 m vs 10.2 m, HR 0.48, CI95 [0.29 × 10^0.81^])	IMpower 131	[86]
BREAST	Atezolizumab PD-L1 + nab-PTX	Atezolizumab	III; previously untreated metastatic TNBC	902	PFS: 7.2 months in the atezolizumab + nab-PTX group vs. 5.5 months in the placebo + nab-PTX group. In PD-L1 + tumors, PFS: 7.5 and 5.0 months, respectively. OS: 21.3 months in the atezolizumab + nab-PTX group vs. 17.6 months in the placebo + nab-PTX group; In PD-L1 + tumors, OS: 25.0 and 15.5 months, respectively.	Clinically meaningful OS benefit observed in PD-L1+ pts (7.5-months median OS improvement). A + nP safe and tolerable	NCT02425891 (IMpassion130)	[88]
BREAST	Atezolizumab PD-L1 + nab-PTX-anthracycline as neoadjuvant for early-stage TNBC	Atezolizumab	III; Early TNBC	455	The pCR was documented with 58% in the atezolizumab + chemotherapy group versus 41% in the placebo + chemotherapy group; in the PD-L1 + population, the pCR was 69% and 49%, respectively.	Significantly improved pathological complete response rates with an acceptable safety profile.	NCT03197935 IMpassion031	[89]
BREAST	Atezolizumab PD-L1 + cobimetinib and PTX	Atezolizumab	II; locally advanced or metastatic TNBC	106	median PFS 5.5 months in the cobimetinib + PTX group versus 3.8 months in the placebo + PTX group.	Cobimetinib +PTX increased PFS and ORR. A + cobi + taxane did not increase ORR. Safety profiles.	NCT02322814 (COLET)	[90]
BREAST	Durvalumab + Anthracycline/ taxane	Durvalumab	II	117	PCR (53.4%, Durvalumab; 44.2%, placebo), OR = 1.45. Durvalumab effect only in cohort (pCR 61.0% vs. 41.4%, OR = 2.22) 0.47% thyroid dysfunction.	The combination increases pCR rate in durvalumab alone. Increased pCR with higher sTILs. Trend for increased PCR rates in PD-L1C tumors.	NCT02685059	[91]
BREAST HER-2-TARGETED	Pembrolizumab+ Trastuzumab	Pembrolizumab	I/II	58	OR (15%, PD-1^+^; 0%, PD-1^−^). fatigue 21%	Safe; with activity and durable clinical benefit in PD-L1+/HER2+, trastuzumab-resistant, advanced BC patients.	NCT02129556 PANACEA	[92]
PANCREAS	Pembrolizumab + Gemcitabine + Nab-paclitaxel	Pembrolizumab	Ib/II; Metastatic	17	PFS: 9.1 months. OS: 15.0 months	70.6% (12/17)	NCT02331251	[94]
PANCREAS	Ipilimumab + Gemcitabine	Ipilimumab	Ib	21	21 ORR: 14% (3/21). PFS: 2.78 months. OS: 6.90 months	Safe and tolerable regimen for PDAC with a similar response rate to gemcitabine alone. 76.2% (16/21) elevated ALT, diarrhea, mostly hematologic toxicity	NCT01473940	[95]
PANCREAS	Nivolumab + Gemcitabine + Nab-paclitaxel		I; Locally advanced or metastatic	50	ORR: 18%. PFS: 5.5 months. OS: 9.9 months	36.0% (18/50; peripheral neuropathy, hypokalemia, diarrhea, increased AST/ALT, mostly hematologic toxicity)	NCT02309177	[96]
GASTRIC	Nivolumab + SOX and nivolumab + Cape/OX)	Nivolumab	II	40	median PFS 9.7 months and 10.6 months (5.6–12.5)	well tolerated. encouraging efficacy for unresectable advanced or recurrent HER2-negative G/GEJ cancer.	ATTRACTION-4 NCT02746796	[97]
GASTRIC	Nivolumab + Cape/Oxa or Folfox	Nivolumab	III	1581	Significant improvements in OS (hazard ratio [HR] 0.71 [98.4% CI 0.59–0.86]; *p* < 0.0001) and PFS (HR 0.68 [98% CI 0.56–0.81]	Nivolumab is the first PD-1 inhibitor to show superior OS, PFS benefit; acceptable safety profile in combination	CheckMate 649 NCT02872116	[98]
GASTRIC	Pembrolizumab + chemotherapy (DDP, 5FU or Cape)	Pembrolizumab	II; previously untreated advanced gastric/gastroesophageal junction adenocarcinoma	56	ORR 60.0% [95% confidence interval (CI), 38.7–78.9] (combination therapy) and 25.8% (95% CI 11.9–44.6) (monotherapy)	Pembrolizumab: antitumor activity; well tolerated. in combination with chemotherapy in patients	KEYNOTE-059 NCT02335411	[99]
PROSTATE	Nivolumab + docetaxel	Nivolumab	II mCRPC in progression after second-generation hormonal therapy and CT-naïve	41	ORR was 36.8% in patients with measurable disease, PSA RR was 46.3%, and rPFS was 8.2 months	Clinical activity in patients with chemotherapy-naïve mCRPC. Safety consistent with the individual components.	CheckMate 9KD; NCT03338790	[102]
PROSTATE	Pembrolizumab + docetaxel	Pembrolizumab	Ib/II	104 mCRPC after second-generation hormonal therapy and CT-naïve	ORR was 18%, PSA RR was 28%, rPFS was 8.3 months, and OS was 20.4 months		KEYNOTE-365	[103]
PROSTATE	Pembrolizumab Plus Docetaxel and Prednisone	Pembrolizumab	Ib/II	104 mCRPC	PSA response rate 34%; ORR (RECIST v1.1) was 23%. Median rPFS and OS 8.5 months and 20.2 months, respectively.	Antitumor activity in chemotherapy-naïve patients with mCRPC treated with abiraterone or enzalutamide for mCRPC. Safety consistent with individual agent profiles	KEYNOTE-365, (Cohort B); NCT02861573	[104]
OVARIAN	Nivolumab + Bevacizumab (ICB + antiangiogenesis)	Nivolumab	II; Recurrent EOC (PSOC + PROC) PSOC PROC	38	ORR %: 40.0% in platinum-sensitive and 16.7% (95% CI 3.6–41.4%) platinum-resistant; mPFS, months: 8.1.	activity in patients with relapsed ovarian cancer; greater activity in platinum-sensitive setting, limited in platinum-resistant patients	NIVO-BEV	[105]

Abbreviations: OS—overall survival (median); PFS—progression free survival (median); GV1001—telomerase reverse transcriptase catalytic subunit class II 16 mer peptide vaccine; GVAX—granulocyte-macrophage colony-stimulating factor-transfected pancreatic tumor vaccine; Cy—cyclophosphamide; CRS-207—live attenuated listeria monocytogenes expressing mesothelin; PD-1—programmed cell death 1; ALT—alternative lengthening of telomeres; AST—aspartate aminotransferase; CAR—chimeric antigen receptor; CTLA-4—cytotoxic T lymphocyte-associated protein 4; MSI-h—microsatellite instability-high; PD-L1—programmed cell death ligand 1. Cape—capecitabine; DDP—cisplatin; HER2—human epidermal growth factor receptor 2; mOS—median OS; mPFS—median PFS; CAPOX—capecitabine plus oxaliplatin; FP—5-Fu plus cisplatin; bev—bevacizumab; BRCAm—BRCA mutated; BRCAwt—BRCA wild-type; C/P—carboplatin and paclitaxel; CTLA-4—cytotoxic T-lymphocyte-associated antigen 4; EOC—epithelial ovarian cancer; HR—hazard ratio; ICI—immune checkpoint inhibitor; ITT—intent to treat; mPFS—median progression-free survival; NR—not reported; NSS—not statistically significant; ORR—overall response rate; PARPi—poly(ADP-ribose) polymerase inhibitor; PLD—pegylated liposomal doxorubicin; PROC—platinum-resistant ovarian cancer; PR-ROC—platinum-resistant recurrent ovarian cancer; PSOC—platinum-sensitive ovarian cancer; y—year; TNBC—triple-negative breast cancer.

**Table 2:** Selected clinical trials providing information on ICI combination with PARP inhibitors in different tumor types.

Tumor Type	Combined Therapy	Anti-PD-1/PD-L1	Clinical Phase and Population	N of Patients	Result	Conclusion	NCT Number	Reference
PROSTATE	Pembrolizumab + Olaparib (Anti-PD1/anti-PDL1 + PARPi)	Pembrolizumab	Ib/II mCRPC progressed to docetaxel and second-generation hormonal therapies	84	PSA RR of 9%, ORR of 8.3%, rPFS of 4 months and OS of 14 months	Additional follow-up: combination still active in docetaxel-pretreated pts. Combination safety consistent with individual profiles of each agent.	KEYNOTE-365 (cohort A)	[106]
PROSTATE	Durvalumab + Olaparib (Anti-PD1/anti-PDL1 + PARPi)	Durvalumab	II Metastatic mCRPC after progression to abiraterone and/or enzalutamide	17	rPFS 16.1 months with 53% serological or radiographic response. rPFS 16.1 months in DNA repair genes alterations and ORR of 83%. Biomarkers: MLH1, PMS2, MSH2, MSH6	Acceptable toxicity and efficacy, particularly in men with DDR abnormalities. Identified biomarkers: MLH1, PMS2, MSH2, MSH6	NCT02484404	[107]
PROSTATE	Nivolumab + ipilimumab (Anti-PD1 + anti-CTLA4)	Nivolumab + ipilimumab	II Metastatic mCRPC	7/44 (16%)	25% ORR in pre-chemotherapy cohort 1 and 10% ORR in post-chemotherapy cohort2. 5.5 and 3.8 months median rPFS and 19.0 and 15.2 months median OS.	Identified potential biomarkers of response: BRCA2, FANCA, ATRX, ERCC3, MLH1, XRCC2	NCT02985957	[108]
BREAST	Pembrolizumab PD-1 + niraparib	Pembrolizumab	II advanced or metastatic TNBC	55	ORR 21% in the pembrolizumab + niraparib group; 47%. in *BRCA* -mutated tumors.	higher response rates in BC with tumor BRCA mutations	NCT02657889	[109]
BREAST	Camrelizumab + apatinib	Camrelizumab	II advanced TNBC	40	ORR 43.3% in the continuous dosing cohort, no objective response in the intermittent dosing cohort.	ORR dramatically higher than previously reported ORR by anti-PD-1/PD-L1 antibody or apatinib monotherapy. Favorable therapeutic effects and a manageable safety profile.	NCT03394287	[110]
OVARIAN	Anti-PD1 (pembrolizumab) + ICB + PARPi (niraparib)	Pembrolizumab	Metastatic OC I/II	20/39 (51%)	Mutational signature 3 correlates with clinical benefit. mutations assessed in BRCA1, BRCA2	Response dependent on interactions of exhausted CD8 + T cells and PD-L1 + macrophages and PD-L1 + tumor cells	NCT02657889	[112]
OVARIAN	ICB + PARPi Pembrolizumab + Niraparib	Pembrolizumab	I-II	62, PR-ROC	ORR 25% DCR 68% In BRCAm: ORR 45%, DCR 73%	Grade 3 TRAE occurred in 16 patients (30%), anemia in 21%, thrombocytopenia (9%)	TOPACIO/Keynote-162 NCT02657889	[11]
OVARIAN	ICB + PARPi Durvalumab and olaparib	Durvalumab	II An uMbrella Study of BIomarker-driven Targeted Therapy	70 PR-ROC	77% overall response rate (ORR) in gBRCA-mutant patients and a 34% ORR in platinum- sensitive BRCA- wild- type patients (*n* = 23/32)	Clinical utility with biomarker-driven targeted therapy. All treatment combinations were manageable, and without unexpected toxicities.	NCT03699449 (AMBITION)	[114]
OVARIAN	ICB + PARPi + anti-angiogenesis. Triplet combination Durvalumab + bevacizumab + olaparib	Durvalumab	I/II Recurrent PSOC: *BRCA*wt *BRCA*m	32 PS ROC with a germline BRCA1/2 mutation	ORR%: BRCAwt: 31.3 BRCAm: 72 DCR of 77.4% (90% CI 61.7–88.9) versus 28.1% (90% CI 15.5–43.9), respectively. The ORR was 87.1% (95% CI 70.2–96.4) versus 34.4% (95% CI 18.6–53.2). The median PFS was 14.7 months (95% CI 10.0–18.1)	Triplet superior over the doublet for all the endpoints. Genomic instability did not correlate with response	MEDIOLA NCT02734004	[115]
OVARIAN	Dostarlimab + niraparib + bevacizumab (Triplet combination)	Dostarlimab	II	41 Patients PR ROC	PFS months (95% CI) 7.6 (4.2–10.6) ORR (%): 17.9 Partial responce	ORR of 47.5% and a clinical benefit rate of 95.0%. Durable responses of longer than 12 months were observed in 25%	OPAL trial NCT03574779	[116]

Abbreviations: OS—overall survival (median); PFS—progression free survival (median); GV1001—telomerase reverse transcriptase catalytic subunit class II 16 mer peptide vaccine; GVAX—granulocyte-macrophage colony-stimulating factor-transfected pancreatic tumor vaccine; Cy—cyclophosphamide; CRS-207—live attenuated listeria monocytogenes expressing mesothelin; PD-1—programmed cell death 1; ALT—alternative lengthening of telomeres; AST—aspartate aminotransferase; CAR—chimeric antigen receptor; CTLA-4—cytotoxic T lymphocyte-associated protein 4; MSI-h—microsatellite instability-high; PD-L1—programmed cell death ligand 1. Cape—capecitabine; DDP—cisplatin; HER2—human epidermal growth factor receptor 2; mOS—median OS; mPFS—median PFS; CAPOX—capecitabine plus oxaliplatin; FP—5-Fu plus cisplatin; bev—bevacizumab; BRCAm—BRCA mutated; BRCAwt—BRCA wild-type; C/P—carboplatin and paclitaxel; CTLA-4—cytotoxic T-lymphocyte-associated antigen 4; EOC—epithelial ovarian cancer; HR—hazard ratio; ICI—immune checkpoint inhibitor; ITT—intent to treat; mPFS—median progression-free survival; NR—not reported; NSS—not statistically significant; ORR—overall response rate; PARPi—poly(ADP-ribose) polymerase inhibitor; PLD—pegylated liposomal doxorubicin; PROC—platinum-resistant ovarian cancer; PR-ROC—platinum-resistant recurrent ovarian cancer; PSOC—platinum-sensitive ovarian cancer; y—year; TNBC—triple-negative breast cancer.

**Table 3:** Selected clinical trials providing information on ICI combination with radionuclide in different tumor types.

Tumor Type	Combined Therapy	Anti-PD-1/PD-L1	Clinical Phase and Population	N of Patients	Result	Conclusion	NCT Number	Reference
PROSTATE	Ipilimumab ± radiotherapy	Ipilimumab	Phase I/II, completed mCRPC	71	16% patients (8/50) had about 50% PSA decline and 1/28 complete response. Grade 3–4 colitis and hepatitis and 1 treatment-related death.	Clinical antitumor activity with disease control and manageable AEs	NCT00323882	[117]
PROSTATE	Atezolizumab + Radium 223	Atezolizumab	Ib mCRPC	44	ORR of 6.8%, PSA RR of 4.5%, and rPFS of 3 months	Low clinical response	NCT02814669	[118]
GMB	Durvalumab and/or bevacizumab + radiotherapy	Durvalumab	II	158 Newly diagnosed MGMT methylated Glioblastoma and recurrent GMB	cohort A (*n* = 40, treatment well-tolerated. mOS:15.1 months, 8 still alive (with survival ranging SR 15.7–34.9 months). cohort B (*n* = 30): OS 59.0% and 44.4% for 6, and 12 months. Post-treatment:partial response in 13.3% of the cohort population (*n* = 4) and none of the patients experienced high-grade treatment-related adverse events (grade 4)	Treatment with RT was well-tolerated. Adding durva to BEV did not improve the outcome of durvalumab alone.	NCT02336165	[119]

Abbreviations: OS—overall survival (median); PFS—progression free survival (median); GV1001—telomerase reverse transcriptase catalytic subunit class II 16 mer peptide vaccine; GVAX—granulocyte-macrophage colony-stimulating factor-transfected pancreatic tumor vaccine; Cy—cyclophosphamide; CRS-207—live attenuated listeria monocytogenes expressing mesothelin; PD-1—programmed cell death 1; ALT—alternative lengthening of telomeres; AST—aspartate aminotransferase; CAR—chimeric antigen receptor; CTLA-4—cytotoxic T lymphocyte-associated protein 4; MSI-h—microsatellite instability-high; PD-L1—programmed cell death ligand 1. Cape—capecitabine; DDP—cisplatin; HER2—human epidermal growth factor receptor 2; mOS—median OS; mPFS—median PFS; CAPOX—capecitabine plus oxaliplatin; FP—5-Fu plus cisplatin; bev—bevacizumab; BRCAm—BRCA mutated; BRCAwt—BRCA wild-type; C/P—carboplatin and paclitaxel; CTLA-4—cytotoxic T-lymphocyte-associated antigen 4; EOC—epithelial ovarian cancer; HR—hazard ratio; ICI—immune checkpoint inhibitor; ITT—intent to treat; mPFS—median progression-free survival; NR—not reported; NSS—not statistically significant; ORR—overall response rate; PARPi—poly(ADP-ribose) polymerase inhibitor; PLD—pegylated liposomal doxorubicin; PROC—platinum-resistant ovarian cancer; PR-ROC—platinum-resistant recurrent ovarian cancer; PSOC—platinum-sensitive ovarian cancer; y—year; TNBC—triple-negative breast cancer.

**Table 4:** Selected clinical trials providing information on ICI combination with vaccines in different tumor types.

Type of Tumor	Combined Therapy	Anti-PD-1/PD-L1	Clinical Phase and Population	N of Patients	Result	Conclusion	NCT Number	Reference
PANCREAS	GVAX + Ipilimumab after FOLFIRINOX vs. FOLFIRINOX continuation	Ipilimumab	II Metastatic	40 vs. 42	PFS: 2.4 mo vs. 5.55 mo. OS: 9.38 mo vs. 14.7 mo) 41.0% (16/39; adrenal insufficiency, hypophysitis, rash, diarrhea)	GVAX and ipilimumab maintenance therapy did not improve OS over continuation of chemotherapy and resulted in a numerically inferior survival in metastatic PDAC		[120]
PANCREAS	Cy/GVAX + CRS-207 vs. CRS-207 vs. Single-agent chemotherapy	Cy/GVAX	IIb Metastatic, previously treated	213 (73 vs. 68 vs. 72)	Median OS in the primary cohort (*n* = 213) was 3.7 (2.9–5.3), 5.4 (4.2–6.4), and 4.6 (4.2–5.7) months in arms A, B, and C, respectively, showing no significant difference between arm A and arm C [*p* = not significant (NS), HR 1.17; 95% CI, 0.84–1.64]	The combination of Cy/GVAX + CRS-207 did not improve survival over chemotherapy	NCT02004262	[121]
PROSTATE	Atezolizumab+ Sipuleucel T	Atezolizumab	Ib	37 mCRPC	ORR 8% after 6 months DCR 41%. rPFS: 8.2 months in arm 1 vs. 5.8 months in arm 2			[122]
PROSTATE	Ipilimumab+ Sipuleucel T	Ipilimumab	III	50 mCRPC	No alteration of antigen-specific responses. Lower baseline frequencies of CTLA-4 expressing T cells and a history of RT.	Modest clinical activity.	NCT01804465	[123]
OVARIAN	Durvalumab + Anti-FRα vaccine	Durvalumab	II	27 Recurrent PROC	Robust FRα-specific T cell responses in all patients	Safe and tolerable. Unexpectedly durable survival in heavily pretreated population.	NCT02764333	[124]
OVARIAN	TILs + ipilimumab + nivolumab	Ipilimumab + nivolumab	I/II	Recurrent EOC	One patient achieved a partial response and 5 others experienced disease stabilization for up to 12 months	improved T cell fold expansion, increased CD8 T cell tumor reactivity, and favorably affect the T cell phenotype	NCT03287674	[125]

Abbreviations: OS—overall survival (median); PFS—progression free survival (median); GV1001—telomerase reverse transcriptase catalytic subunit class II 16 mer peptide vaccine; GVAX—granulocyte-macrophage colony-stimulating factor-transfected pancreatic tumor vaccine; Cy—cyclophosphamide; CRS-207—live attenuated listeria monocytogenes expressing mesothelin; PD-1—programmed cell death 1; ALT—alternative lengthening of telomeres; AST—aspartate aminotransferase; CAR—chimeric antigen receptor; CTLA-4—cytotoxic T lymphocyte-associated protein 4; MSI-h—microsatellite instability-high; PD-L1—programmed cell death ligand 1; Cape—capecitabine; DDP—cisplatin; HER2—human epidermal growth factor receptor 2; mOS—median OS; mPFS—median PFS; CAPOX—capecitabine plus oxaliplatin; FP—5-Fu plus cisplatin; bev—bevacizumab; BRCAm—BRCA mutated; BRCAwt—BRCA wild-type; C/P—carboplatin and paclitaxel; CTLA-4—cytotoxic T-lymphocyte-associated antigen 4; EOC—epithelial ovarian cancer; HR—hazard ratio; ICI—immune checkpoint inhibitor; ITT—intent to treat; mPFS—median progression-free survival; NR—not reported; NSS—not statistically significant; ORR—overall response rate; PARPi—poly(ADP-ribose) polymerase inhibitor; PLD—pegylated liposomal doxorubicin; PROC—platinum-resistant ovarian cancer; PR-ROC—platinum-resistant recurrent ovarian cancer; PSOC—platinum-sensitive ovarian cancer; y—year; TNBC—triple-negative breast cancer.

GVAX is a cell-based vaccine that transfers an allogeneic tumor cell engineered to express granulocyte-macrophage colony stimulating factor (GM-CSF). According to Wu et al., GVAX and ipilimumab after FOLFIRINOX resulted in lower overall survival than continuation of FOLFIRINOX chemotherapy in patients with metastatic pancreatic cancer [120]. CRS-207 is a microorganism-based vaccine that transfers a live-attenuated Listeria monocytogenes (Lm) engineered to express the PDAC-associated antigen mesothelin [121]. The combination of ICI and cancer vaccines has also been evaluated in prostate cancer; in this regard, a phase Ib study has assessed the combination of Sipuleucel T and atezolizumab in two arms of sequential treatment: atezolizumab followed by Sipuleucel T and vice versa. Thirty-seven patients were included, with an ORR after 6 months of 8% and a DCR of 41%. rPFS was 8.2 months in arm 1 vs. 5.8 months in arm 2 [122]. Sipuleucel-T is an autologous cellular immunotherapy that improves survival in patients with metastatic castration-resistant prostate cancer (mCRPC). A total of 50 patients with mCRPC were enrolled in a clinical trial (NCT01804465) where they received ipilimumab either immediately or delayed for 3 weeks following completion of sipuleucel-T treatment [122].

To date, trials testing tumor-infiltrating lymphocytes (TIL) and adoptive cell therapy (ACT) alone in EOC have demonstrated feasibility but modest efficacy. Instead, combining a multi-epitope antifolate receptor (anti-FR) vaccine with durvalumab (NCT02764333) in PROC has been reported to be safe and tolerable [124]. Ipilimumab and nivolumab with adoptive cell therapy were tested in NCT032876, showing that the combination of ICI and ACT is feasible and safe. One partial response and one long-lasting SD demonstrated the potential of ACT in OC.

### 5.5. Selected Clinical Trials Providing Information on ICI Combination with Novel Hormonal Therapies in Different Tumor Types (Table 5)

Immune checkpoint inhibitors and second-generation hormonal treatments have been associated, particularly in PDAC, with resistance to enzalutamide due to an increased expression of PD-L1 in dendritic cells, despite the controversial immunomodulatory role of the new hormonal therapies [126].

The KEYNOTE-199 trial evaluated the combination of enzalutamide and pembrolizumab in two cohorts of patients with mCRPC refractory to enzalutamide (cohort 4: measurable disease; cohort 5: predominantly bone disease) [127]. In cohort C of the phase Ib/II KEYNOTE-365 study, patients with mCRPC that had progressed to abiraterone received enzalutamide plus pembrolizumab. They included 103 patients with a PSA RR of 22%, an ORR of 12%, 10 of 18 patients with measurable disease, and a DCR of 32% [128].

### 5.6. Selected Clinical Trials Providing Information on ICI Combination with CAR-T Cell Immunotherapy in Different Tumor Types (Table 6)

Adoptive CAR (chimeric antigen receptor) T cell therapy is the ex vivo expansion of a patient’s T cells that have been genetically engineered to express a CAR that recognizes a particular tumor antigen. To date, no CAR T cell therapy has been approved to treat solid malignancies.

Regarding PDAC, few clinical trials have demonstrated limited efficacy, and EGFR is expressed by a wide range of tissues, which may explain the high incidence of TRAEs. Despite this, based on promising preclinical mouse studies, trials are trying to elucidate the clinical outcomes of anti-mesothelin CAR T cell therapy. For example, a phase 1 trial (NCT01897415) showed that among *n* = 6 patients with aPDAC, none experienced severe TRAEs. *n* = 2 (33.3%) reached SD [129]. As for PDAC, currently, several barriers still limit the success of CAR T cell therapies in EOC and other solid tumors, including the limited number and heterogeneous expression of membrane antigen targets, inadequate tracking of T cells to tumor sites, and limited fitness and survival in the TME. For example, mesothelin-directed CAR T cells with CRISPR-Cas9-mediated knockout of PD-1 are being tested in mesothelin-positive solid tumors, including EOC.

A pilot dose escalation study to investigate mesothelin-directed CAR-T cells with only PD-1 disruption by CRISPR (termed as GC008t) in patients with mesothelin-positive advanced solid tumors (NCT03747965). Nine patients (6 pancreatic cancers, 2 ovarian cancers, and 1 colorectal cancer) were treated. Eight of the nine patients received a lymphodepletion regimen of cyclophosphamide and nab-paclitaxel with or without gemcitabine. In addition, four of the nine patients received repeat infusions of GC008t per protocol. This Phase I trial of GC008t further establishes that genetic inactivation of PD-1 in CAR-T cells by CRISPR is feasible and safe [130]. Similar results were obtained in another phase I study in fifteen enrolled adult patients with measurable MSLN+ (≥10% of tumor cells expressing MSLN) locally advanced or metastatic solid tumors who failed at least one standard therapy or were unable to tolerate chemotherapy [131].

### 5.7. Selected Clinical Trials Providing Information on ICI Combination with Viral and other Therapies in Different Tumor Types (Table 7)

Viral therapies: In a study about viral-based therapies in GMB tumors, the CAPTIVE/KEYNOTE-192 study enrolled 49 participants with recurrent HGG who underwent intratumoral administration of DNX-2401 and sequential adjuvant pembrolizumab therapy [132].

Targeting ROS: The level of ROS in cancer cells is typically higher than in their normal surrounding cells. This level can be modulated by combining chemo/radiotherapy and immunotherapy. The work by Fundora Ramos et al. [133] was conducted to identify the efficacy and safety of Oncoxin-Viusid (OV) as a supportive treatment for patients with prostate cancer (PCA). A prospective, non-randomized, open-label phase II clinical trial, including 25 patients with hormone-refractory PCA (HRPC).

**Table 5:** Selected clinical trials providing information on ICI combination with novel hormonal therapies in different tumor types.

Tumor Type	Combined Therapy	Anti-PD-1/PD-L1	Clinical Phase and Population	N of Patients	Result	Conclusion	NCT Number	Reference
PROSTATE	Pembrolizumab (immune checkpoint blockade) + enzalutamide (androgen receptor inhibitor)	Pembrolizumab	mCRPC refractory to enzalutamide	126	In cohort 4: 12% had a response, 51% disease control rate (DCR). In cohort 5, 51% DCR. rPFS: 4 months in both cohorts. All Grade (C4 75%, C5 69%) and Grade 3–5 (C4 26%, C5 24%) adverse events (AEs) were similar as compared to cohorts 1–3 but numerically more frequent	Data demonstrate clinical support the addition of enzalutamide to with pembrolizumab.	KEYNOTE-199	[127]
PROSTATE	Pembrolizumab + enzalutamide	Pembrolizumab	Ib/II mCRPC that had progressed to abiraterone	103	PSA RR 22%, ORR 12% DCR of 32%	Pembro + enza continued to show activity in pts with abi-pretreated mCRPC. Safety was consistent with the known profiles of pembro and enza.	KEYNOTE-365	[128]

Abbreviations: OS—overall survival (median); PFS—progression free survival (median); GV1001—telomerase reverse transcriptase catalytic subunit class II 16 mer peptide vaccine; GVAX—granulocyte-macrophage colony-stimulating factor-transfected pancreatic tumor vaccine; Cy—cyclophosphamide; CRS-207—live attenuated listeria monocytogenes expressing mesothelin; PD-1—programmed cell death 1; ALT—alternative lengthening of telomeres; AST—aspartate aminotransferase; CAR—chimeric antigen receptor; CTLA-4—cytotoxic T lymphocyte-associated protein 4; MSI-h—microsatellite instability-high; PD-L1—programmed cell death ligand 1; Cape—capecitabine; DDP—cisplatin; HER2—human epidermal growth factor receptor 2; mOS—median OS; mPFS—median PFS; CAPOX—capecitabine plus oxaliplatin; FP—5-Fu plus cisplatin; bev—bevacizumab; BRCAm—BRCA mutated; BRCAwt—BRCA wild-type; C/P—carboplatin and paclitaxel; CTLA-4—cytotoxic T-lymphocyte-associated antigen 4; EOC—epithelial ovarian cancer; HR—hazard ratio; ICI—immune checkpoint inhibitor; ITT—intent to treat; mPFS—median progression-free survival; NR—not reported; NSS—not statistically significant; ORR—overall response rate; PARPi—poly(ADP-ribose) polymerase inhibitor; PLD—pegylated liposomal doxorubicin; PROC—platinum-resistant ovarian cancer; PR-ROC—platinum-resistant recurrent ovarian cancer; PSOC—platinum-sensitive ovarian cancer; y—year; TNBC—triple-negative breast cancer.

**Table 6:** Selected clinical trials providing information on ICI combination with CAR-T cell immunotherapy in different tumor types.

Tumor Type	Combined Therapy	Anti-PD-1/PD-L1	Clinical Phase and Population	N of Patients	Result	Conclusion	NCT Number	Reference
PANCREAS	Mesothelin-specific		I	6 Metastatic	SD: stabilized disease: 2 patients (33%) with PFS of 3.8 and 5.4 mo No severe TRAEs	Evidence for the potential antitumor activity of messenger RNA CARTmeso cells, as well as PDAC resistance to the immune response		[129]
PANCREAS OVARIAN	Knocked-out PD-1, mesothelin-directed CAR Ts (GC008t)	Mesothelin-positive solid tumors including EOC	I Mesothelin-positive solid tumors including EOC	9 Mesothelin-positive solid tumors including EOC	The best response of the 7 evaluable patients was stable disease in 4 and partial response in 2 patients (dosed ≥ 1 × 10^7^/kg) with PFS of 80 and 160 days.	genetic inactivation of PD-1 in CAR-T cells by CRISPR is feasible and safe	NCT03747965	[130]
Solid tumors	PD-1 and T cell receptor (TCR) deficient mesothelin-specific CAR-T (MPTK-CAR-T	Mesothelin-positive solid tumors	I	15	The best overall response was stable disease (2/15 patients). No dose-limiting toxicity or unexpected adverse events were observed in any of the 15 patients	Feasibility and safety of CRISPR-engineered CAR-T cells with PD-1 disruption		[131]

Abbreviations: OS—overall survival (median); PFS—progression free survival (median); GV1001—telomerase reverse transcriptase catalytic subunit class II 16 mer peptide vaccine; GVAX—granulocyte-macrophage colony-stimulating factor-transfected pancreatic tumor vaccine; Cy—cyclophosphamide; CRS-207—live attenuated Listeria monocytogenes expressing mesothelin; PD-1—programmed cell death 1; ALT—alternative lengthening of telomeres; AST—aspartate aminotransferase; CAR—chimeric antigen receptor; CTLA-4—cytotoxic T lymphocyte-associated protein 4; MSI-h—microsatellite instability-high; PD-L1—programmed cell death ligand 1; Cape—capecitabine; DDP—cisplatin; HER2—human epidermal growth factor receptor 2; mOS—median OS; mPFS—median PFS; CAPOX—capecitabine plus oxaliplatin; FP—5-Fu plus cisplatin; bev—bevacizumab; BRCAm—BRCA mutated; BRCAwt—BRCA wild-type; C/P—carboplatin and paclitaxel; CTLA-4—cytotoxic T-lymphocyte-associated antigen 4; EOC—epithelial ovarian cancer; HR—hazard ratio; ICI—immune checkpoint inhibitor; ITT—intent to treat; mPFS—median progression-free survival; NR—not reported; NSS—not statistically significant; ORR—overall response rate; PARPi—poly(ADP-ribose) polymerase inhibitor; PLD—pegylated liposomal doxorubicin; PROC—platinum-resistant ovarian cancer; PR-ROC—platinum-resistant recurrent ovarian cancer; PSOC—platinum-sensitive ovarian cancer; y—year; TNBC—triple-negative breast cancer.

**Table 7:** Selected clinical trials providing information on ICI combination with viral and other therapies in different tumor types.

Tumor Type	Combined Therapy	Anti-PD-1/PD-L1	Clinical Phase and Population	N of Patients	Result	Conclusion	NCT Number	Reference
GMB	Pembrolizumab+ DNX-2401 oncolytic adenovirus. Intratumoral administration of DNX-2401 and sequential, adjuvant pembrolizumab b	Pembrolizumab	II Open label single group study	49 Recurrent glioblastoma or gliosarcoma	>94% tumor regression.12 months mOS in 48 patients	safe without any dose-limiting conditions	NCT02798406 (CAPTIVE/KEYNOTE-192)	[132]
Targeting ROS								
PROSTATE	Docetaxel + OV Docetaxel + Radiotherapy + OV		II	25 Hormone refractory	Oncoxin-Viusid (OV) −75 mL/day Suppresses ROS production PFS 59% OS rate 64% at 1 year	Clinical and humoral response, high survival rates, delayed appearance of signs of disease progression.	NCT03543670	[133]

Abbreviations: OS—overall survival (median); PFS—progression free survival (median); GV1001—telomerase reverse transcriptase catalytic subunit class II 16 mer peptide vaccine; GVAX—granulocyte-macrophage colony-stimulating factor-transfected pancreatic tumor vaccine; Cy—cyclophosphamide; CRS-207—live attenuated listeria monocytogenes expressing mesothelin; PD-1—programmed cell death 1; ALT—alternative lengthening of telomeres; AST—aspartate aminotransferase; CAR—chimeric antigen receptor; CTLA-4—cytotoxic T lymphocyte-associated protein 4; MSI-h—microsatellite instability-high; PD-L1—programmed cell death ligand 1; Cape—capecitabine; DDP—cisplatin; HER2—human epidermal growth factor receptor 2; mOS—median OS; mPFS—median PFS; CAPOX—capecitabine plus oxaliplatin; FP—5-Fu plus cisplatin; bev—bevacizumab; BRCAm—BRCA mutated; BRCAwt—BRCA wild-type; C/P—carboplatin and paclitaxel; CTLA-4—cytotoxic T-lymphocyte-associated antigen 4; EOC—epithelial ovarian cancer; HR—hazard ratio; ICI—immune checkpoint inhibitor; ITT—intent to treat; mPFS—median progression-free survival; NR—not reported; NSS—not statistically significant; ORR—overall response rate; PARPi—poly(ADP-ribose) polymerase inhibitor; PLD—pegylated liposomal doxorubicin; PROC—platinum-resistant ovarian cancer; PR-ROC—platinum-resistant recurrent ovarian cancer; PSOC—platinum-sensitive ovarian cancer; y—year; TNBC—triple-negative breast cancer.

## 6. The Immunotherapy Side Effects: Focus on Cardiac Toxicity

Compared with other types of cancer therapy, the timing of side effects related to immunosuppressants is less predictable. In patients receiving immunotherapies, side effects may occur shortly after the first dose of a medicine or long after treatment has stopped. Because cancer immunotherapy drugs are relatively new, there is limited evidence from clinical trials about managing treatment-related side effects. To address this lack of knowledge, the American Society for Clinical Oncology and the National Comprehensive Cancer Network jointly developed guidelines on how physicians should manage complications associated with checkpoint inhibitors in 2018 [134]. Guidelines on when to use steroids and when to stop immunosuppressant therapy are included. Although many questions concerning managing immune side effects remain unresolved, experts have agreed that it is important to detect them before they evolve into more severe complications.

Major adverse events of CAR T cell immunotherapy include cytokine release syndrome (CRS) and neurological toxicity [135]. CRS is a disease characterized by fever, hypotension, and acute hypokalemia due to supraphysiological cytokine levels, including IL-6, TNF-α, IL-10, and IFN-Υ, in activated CAR T lymphocytes or other immunocompromised cells such as macrophages [136]. This is a systemic inflammatory disorder with multiorgan involvement that ranges from mild to severe. Typically, the grading scale uses fever, hypotension, and hypoxia as components of the severity assessment. Several grading systems have been used over the past years, but the most commonly used is provided by the American Society for Transplantation and Cellular Therapy (ASTCT) [136] and the Common Terminology Criteria for Adverse Events (CTCAE) [137]. Novel grading systems continue to evolve, including the so-called CARTOX system (CAR-T cell therapy-associated TOXicity), and their applicability requires further characterization [138].

Finally, the severity of CRS coincides with the disease burden and the higher infused CAR T cell dose. In most cases, the effects of CART cell therapy on cardiovascular disease (CV) have been reported in conjunction with CRS. Major adverse cardiac events (MACE) associated with CRS include de novo arrhythmia, left ventricular (LV) dysfunction, elevated troponin, cardiomyopathy, heart failure, and sudden cardiac death [139,140,141].

Although it is impossible to rule out the development of separate CV toxicities such as sinus tachycardia and arterial hypotension, they can be seen primarily as a consequence of CRS. Usually, the median time to CRS was 5–6 days, and the median time to MACE was 11–21 days after CAR T cell infusion. Based on registry cohorts, the patients who developed cardiomyopathy were older and had a higher prevalence of CV risk factors. Although left ventricular ejection fraction often recovered, many patients demonstrated persistent cardiac dysfunction [142]. Although the pathophysiology of CAR T cell therapy-induced CV adverse events is not fully understood, it is thought to be similar to cardiomyopathy associated with sepsis and stress, likely through IL-6, which has been implicated as a mediator of myocardial depression in infectious and inflammatory states [143]. There is still no established best preventive strategy for reducing CV complications. A practical approach to treating patients who have received CAR T cell therapy involves carefully assessing CV symptoms with targeted use of serum biomarkers and imaging parameters to detect cardiotoxicity early while initiating supportive treatment as appropriate [144]. CAR T cell-related cardiotoxicity data are currently limited to retrospective registry cohorts, which further need to be investigated to provide evidence-based guidance on the effective use of CART cell therapy and optimizing CV outcomes. Efficient management of CV adverse reactions while maintaining anticancer efficacy will be a crucial challenge in cardio-oncology, as seen from the entire area of immune-oncology.

## 7. Challenges in Immunotherapy

The scientific community believes the capacity to build new therapies that benefit more patients is coming soon. Despite several molecular targets being undruggable yet, it is expected that several fields of fundamental research will strikingly spread out soon, helping to face the issue, including gene editing, structural biology for protein design, medicinal chemistry, epigenetics, and imaging technologies.

The field of engineered cell therapies, including CAR T cells, T cells generated by lentivirus transfection (TCR T cells), and Bispecific T cells (BiTEs), is advancing very quickly. Although it is still the beginning, the technologies are exploding, and we can now modify the immune cells to improve their function and survival after infusion into patients, improving their effectiveness [145,146].

It is important to point out that this strategy can be effective even in patients whose immune systems are not responding to the target antigens already encountered. We can now better dissect the cell composition of the tumor microenvironment (TME) at the single-cell level, and by using multi-omic strategies, including spatial transcriptomics [147], we can identify the inhibitory signals that hamper immune function. Thanks to these advances, we can turn those inhibitory signals into positive ones by engineering the immune cells [145,146].

Furthermore, these advances, coupled with improved selectivity for specific tumor targets, will have an extraordinary impact on developing effective therapies against solid tumors, which is still a challenge [148]. We already have several ongoing clinical trials targeting solid tumors (Table 1, Table 2, Table 3, Table 4, Table 5, Table 6 and Table 7). Another area that is expected to advance soon is the development of drugs targeting inhibitory molecules in the tumor microenvironment (TME). It is reasonable that increasing numbers of those drugs will be tested in clinical trials, especially as combination therapies [149,150]. Within the area of cancer immunotherapy, it is predicted that there will be a propagation of combinatorial strategies by using combination agents that target independent cellular pathways, leading to synergistic effects [151,152] (Table 1, Table 2, Table 3, Table 4, Table 5, Table 6 and Table 7). In addition to well-known independent cellular pathways that can be targets in a combination strategy with immunotherapy, we also discuss emerging pathways such as oxidative stress (ROS levels) and ubiquitin ligase as regulators of tumor immune response (Table 7). Oncology research is expected to see progress in cancer vaccines as well. The vaccine evolution might have the power to overcome some of the challenges that still impact this approach, and it is close to reaching clinical use [153]. The mRNA vaccine technology, which works so effectively against COVID-19, was developed years ago for cancer vaccines, although the initial efforts were minimally successful [153,154]. The other success of the COVID-19 vaccines versus tumor vaccines has highlighted the critical divergence between developing a vaccine targeting a tumor that already exists in the body and is not being effectively recognized by the immune system versus a pathogen that has a genome utterly different from the human genome.

Despite the initial lack of success of cancer vaccines, a large amount of information coming from essential studies will be helpful to explain the failure of more robust responses. In addition, it will help overcome some obstacles and yield a more effective vaccine. Notably, through deep sequencing, it is now possible to identify the patient’s cancer unicity rapidly and efficiently, helping to create personalized vaccines that exploit this uniqueness [155]. The critical challenge now is teaching the immune system to respond to these vaccines because the tumor has already elaborated strategies to switch off and evade the immune response. In the future, cancer vaccines will be crucial and will be integrated into cancer therapies.

## 8. Conclusions

The major outstanding questions and directions for future research are described below. Although it is a time for optimism in cancer immunotherapy, several exceptional questions remain unanswered.

### 8.1. What Are the Tumor-Specific and/or Tumor-Agnostic Biomarkers for Response to Immunotherapy? Despite the Many Options Available for Immunotherapy Strategies, How do We Choose which One Deserves Priority for Clinical Trials? (Figure 3)

Although several predictive biomarkers have been widely investigated for immunotherapy response [156], currently only a few tumor-agnostic biomarkers are FDA-approved [157,158]. Furthermore, despite the many options available for immunotherapy, there is still no evidence to help us choose which of these deserves to be used in clinical trials over another.

**Figure 3:**
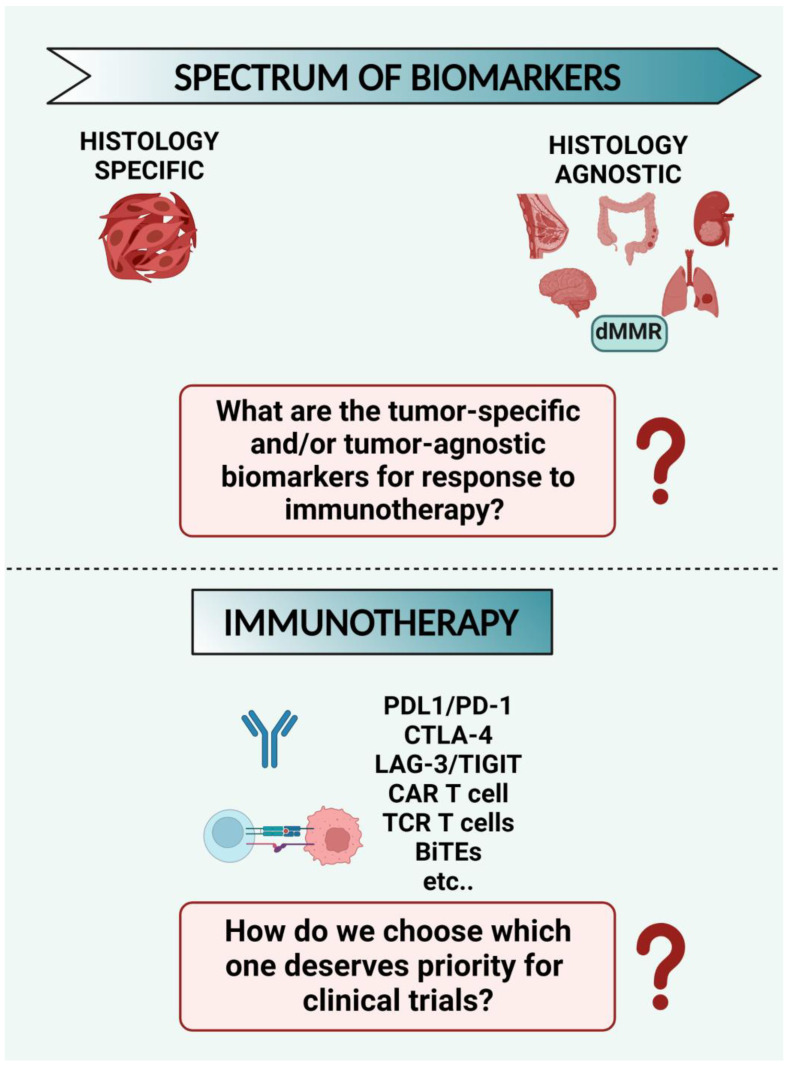
General scheme of biomarkers for response to immunotherapy. Several predictive biomarkers have been widely investigated for immunotherapy response, but currently, only a few tumor-agnostic biomarkers are FDA-approved (e.g., mismatch repair deficient tumor, dMMR). Despite many options available for immunotherapy (PDL1/PD-1, CTLA-4, LAG-3/TIGIT, CAR T cells, TCR T cells, BiTEs, etc.), there is still no evidence to help us choose which one deserves priority for clinical trials.

### 8.2. How can we Modulate the Reactive Oxygen Species (ROS Levels) to Improve Immunotherapy Sensitivity? (Figure 4)

ROS modulation is a promising cancer immunotherapy strategy (Figure 1) and deserves in-depth basic research and clinical investigations. To boost ROS-based cancer immunotherapy, several aspects must be investigated, including (i) the cancer-specific redox signaling events in a given type of cancer cell. This approach could allow for choosing ROS-elevating or ROS-depleting therapy specific to particular types of cancer cells; (ii) the ROS contribution to ICIs therapy in controlling immune checkpoints such as PD-(L)1 and CTL-4 expressed on cancer cells in immunosuppressive TME; (iii) the contribution of the ROS-associated drug to the interplay between tumor hypoxia and PD-L1 expression; (iv) the administration of nanoparticles as targeted delivery systems to specifically provide ROS over-generation in immunosuppressive TME without generating side effects. It is believed that a multimodal therapeutic approach involving simultaneous or sequential administration of redox modulators, conventional chemotherapy, and targeted therapies, such as ICIs therapy, holds the highest potential for obtaining a more lasting and safe therapeutic benefit for cancer patients [17].

**Figure 4:**
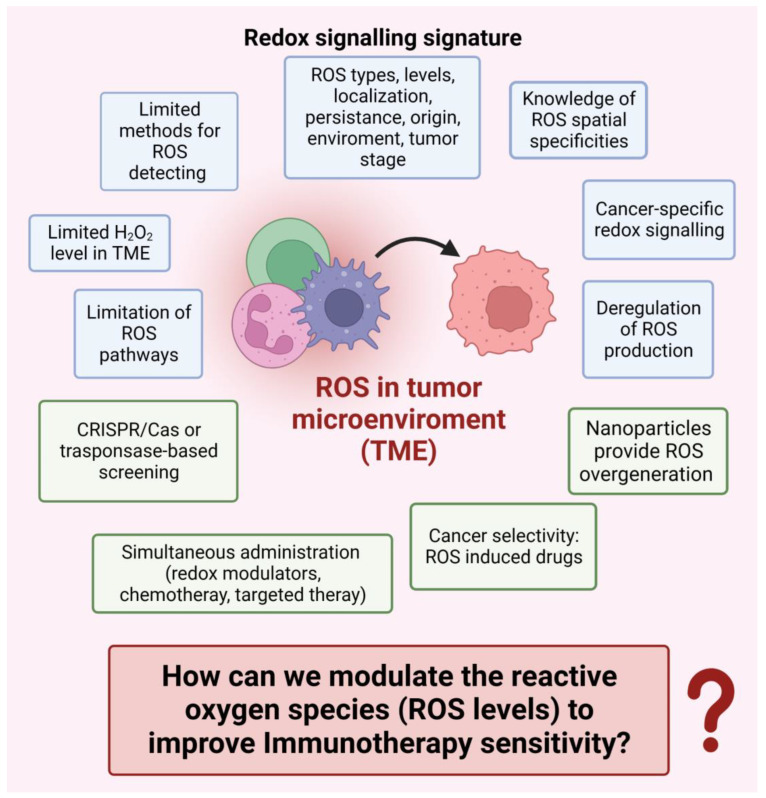
Redox signaling signature in the tumor microenvironment (TME). ROS modulation represents a promising strategy in cancer immunotherapy, but to boost ROS-based cancer immunotherapy, several aspects must be investigated. The cartoon highlights the crucial factors to be considered to exploit ROS to improve immunotherapy sensitivity. ROS—reactive oxygen species; H_2_O_2_—hydrogen peroxide.

### 8.3. What Effect may the Ubiquitin Signaling Modulation have on Immunotherapy Sensitivity? (Figure 5)

Ubiquitin signaling is involved in several aspects of TME, and its dysregulation may contribute to tumor progression. Therefore, targeting ubiquitin signaling can be a promising therapeutic strategy for cancer treatment. Noteworthy, a small molecule named proteolysis targeting chimera (PROTAC) has been approved by the FDA. Although only a few E3 ligases have been used in PROTACs technology, they have several advantages, including good solubility and cell permeability, and are therefore promising for the future. While the specific role of ubiquitin signaling in the immune system is still unclear and the immunotherapies targeting ubiquitination are not fully understood, the interplay of the ubiquitin system and TME deserves more investigations in the future [12].

**Figure 5:**
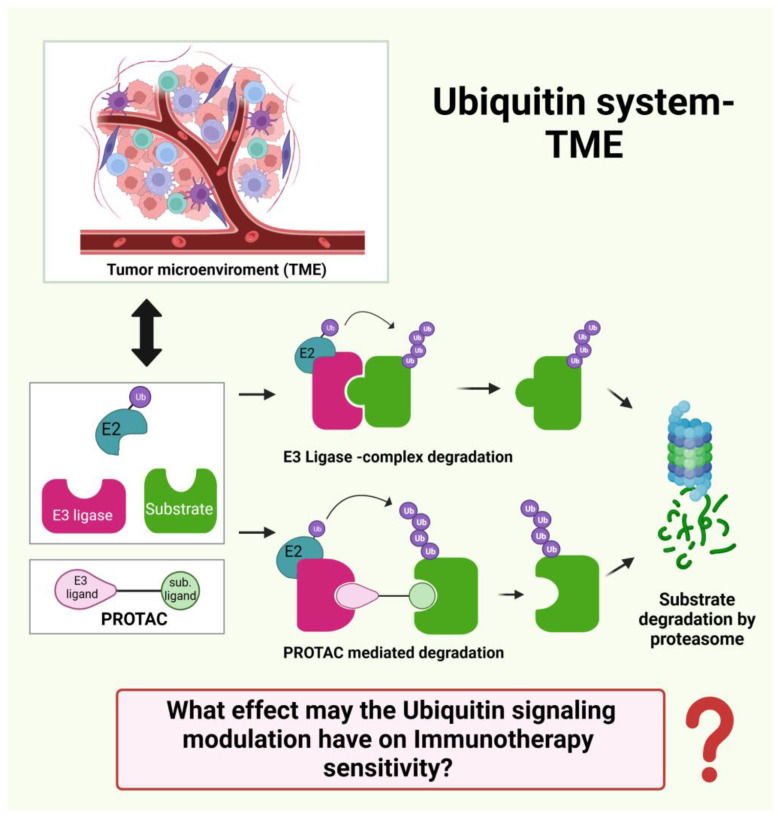
General scheme of ubiquitin signaling–TME interplay. Ubiquitin signaling is involved in several aspects of the tumor microenvironment (TME), and its dysregulation may contribute to tumor progression. Therefore, targeting ubiquitin signaling can be a promising therapeutic strategy for cancer treatment. A small molecule named proteolysis targeting chimera (PROTAC) has been approved by the FDA. These small molecules have several advantages, including good solubility and cell permeability. However, the specific role of ubiquitin signaling in the immune system is still unclear, so the interplay of the ubiquitin system and TME deserves more investigations in the future.

### 8.4. How can Imaging Techniques Be Used in the Diagnostic and Therapeutic Immunotherapy Field? (Figure 6)

(1) Although there are specific proteins that distinguish the surfaces of different extracellular vesicles (EVs),there is still no consensus on which of these proteins can be reliably used as markers for various types of EVs. Flow cytometry techniques have recently been developed to characterize different types of EVs based on their surface markers. However, little is known about the relationship between these surface markers and the molecule cargo carried by EVs, which may reflect the physiological and pathological state of the cells that generated them.

(2) Combining super-resolution localization techniques (STORM, PALM, and PAINT) with techniques that can provide morphological information, such as AFM or TEM, can yield a more complete understanding of the structure and function of biological samples at the nanoscale level. This approach has the potential to advance our understanding of fundamental biological processes and aid in developing new diagnostic and therapeutic strategies for a range of diseases.

**Figure 6:**
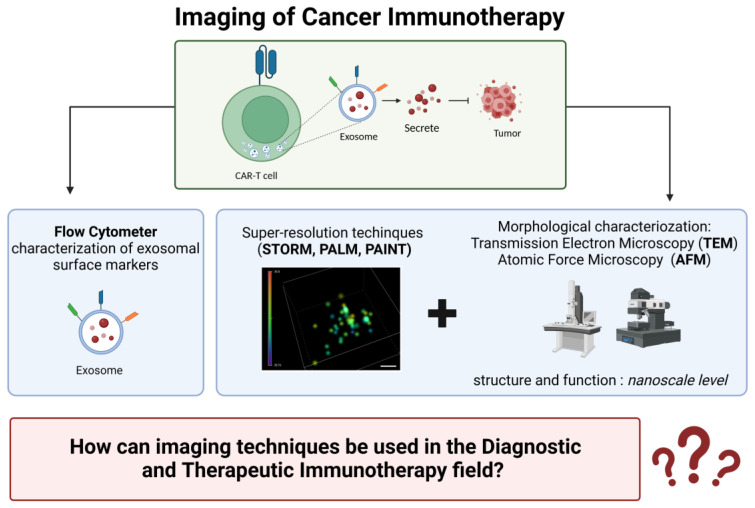
Imaging technology in cancer immunotherapy. Imaging techniques are crucial for monitoring tumor responses during treatment. However, despite the explosion of immune-oncological drugs in the past decade, there are several limitations to this strategy: (1) The development of clinical imaging biomarkers for anti-cancer immunotherapy is not keeping up with the speed of immunotherapy development; (2) the spatial resolution of clinical cameras/scanners depends on the radioisotopes’ positron range; and (3) the need to test new markers on animal models with different genetic makeup and immune systems than humans prevents a clear understanding of the tumor microenvironment at the cellular and subcellular levels. Exosomes are nano-sized extracellular vesicles secreted by most cells in the body and carry a range of functional molecules. For example, exosomes have been found to play a pivotal role in cytokine release in CAR-T cell therapy. Scale bar, 20 nm.

### 8.5. How may the Microbiome Affect Immunotherapy Sensitivity? (Figure 7)

The era of the microbiome is upon us, and several pre-clinical and clinical reports shed light on the unconventional role of the microbiota as a mediator of the response to cancer therapy, including cancer immunotherapy. Critical questions emerged from these studies, including the need for microbiome profiling in patients undergoing cancer therapy (which sequencing methods and which reference databases should we use?).

**Figure 7:**
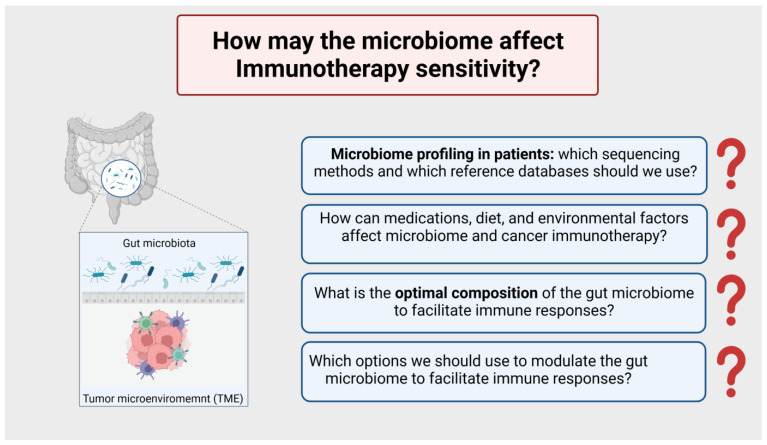
General scheme of the microbiota as a mediator of the response to cancer immunotherapy. Several pre-clinical and clinical studies shed light on the role of the microbiota as a mediator of the response to cancer therapy, including cancer immunotherapy. From these studies emerged critical questions: (1) The need for microbiome profiling in patients in cancer therapy (which sequencing methods and which reference databases should we use?). (2) How medications, diet, and environmental factors (factors affecting the gut microbiome) may affect cancer immunotherapy? (3) What is the optimal composition of the gut microbiome to facilitate immune responses? (4) Which options we should use to modulate the gut microbiome to facilitate immune responses?

Additional questions must be addressed, including how medications, diet, and environmental factors (factors affecting the gut microbiome) may affect cancer immunotherapy. Furthermore, it is not easy to understand the optimal composition of the gut microbiome to facilitate immune responses and which options we should use to modulate them. Only through a deep comprehension of this interplay will it be possible to exploit the gut microbiota’s modulation to enhance the efficacy of immunotherapy [159].

### 8.6. Where Are We about more Effective Cancer Vaccines? (Figure 8)

Still, many challenges need to be faced when making cancer vaccines. Noteworthy, some of those have already been overcome thanks to the discovery of specific tumor antigens and the ability to target them [160]. However, it is still inevitable to face other challenges, such as the time the clinical trial takes to test a vaccine and the time needed to develop vaccines specific to different types of tumors. However, recently, cancer vaccines have made significant advances. The speed-up of mRNA technology development has made the routine vaccine timeline faster and more functional. Despite the fact that a few decades ago, cancer vaccines were only a fantasy, they are now becoming a real science.

**Figure 8:**
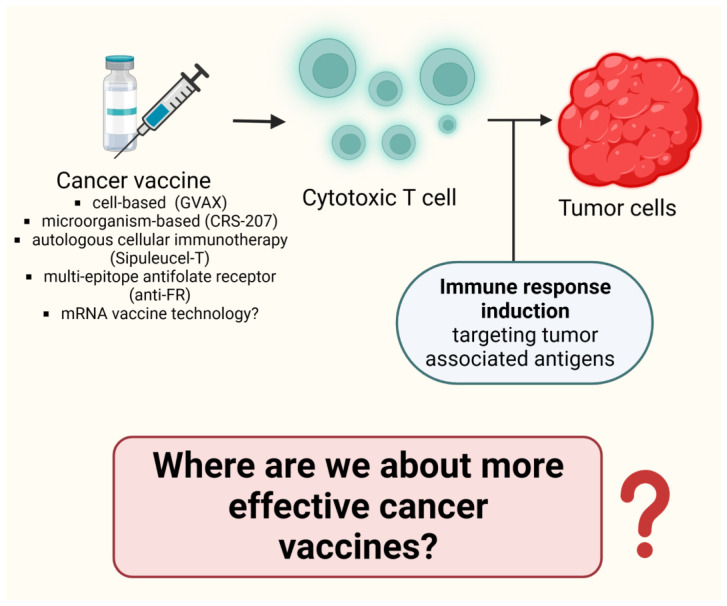
Cancer vaccine: where are we? Many challenges need to be faced when making cancer vaccines. Some challenges have already been overcome thanks to the discovery of specific tumor antigens and the ability to target them. Still, other challenges need to be addressed: (1) the time the clinical trial takes to test a vaccine; (2) the time needed to develop vaccines specific to different types of tumors. Recently, cancer vaccines have made significant advances. The speed-up of mRNA technology development has turned the standard vaccine timeline into one that is faster and more functional.

## Data Availability

Data regarding this study are available upon request to the corresponding authors.
